# Celastrol Attenuates Doxorubicin-Induced Heart Failure by Preserving Mitochondrial Integrity and Suppressing Oxidative Stress and Is Accompanied by Changes in Gut Microbiota

**DOI:** 10.3390/ph19091494

**Published:** 2026-09-20

**Authors:** Yang Zhang, Jingyi Qi, Mingyang Cui, Yufang Shi, Xinyu Luo, Chang Fan, Sitong Wan, Peng An, Yongting Luo, Junjie Luo

**Affiliations:** Department of Nutrition and Health, China Agricultural University, Beijing 100193, China; sy20253314223@cau.edu.cn (Y.Z.); zb20213311055@cau.edu (J.Q.); cuimy_86@cau.edu.cn (M.C.); syff@cau.edu.cn (Y.S.); b20253311588@cau.edu.cn (X.L.); fanchang@cau.edu.cn (C.F.); adeline7wan@cau.edu.cn (S.W.)

**Keywords:** doxorubicin-induced cardiotoxicity, celastrol, mitochondrial function, oxidative stress, gut microbiota

## Abstract

**Background/Objectives:** Doxorubicin (DOX) is an effective chemotherapeutic agent whose clinical use is limited by dose-dependent cardiotoxicity that may progress to irreversible heart failure. Celastrol (Cel), a bioactive triterpenoid from *Tripterygium wilfordii*, has recognized anti-inflammatory and antioxidant activities, but its potential for treating DOX-induced heart failure remains unclear. This study investigated the cardioprotective effects of Cel in a murine model of DOX-induced heart failure. **Methods:** Cardiac function was assessed by echocardiography, while serum biochemical assays, histological staining, transmission electron microscopy, RT-qPCR, ATP measurement, and 16S rRNA sequencing were used to evaluate myocardial injury, oxidative stress, mitochondrial structure and function, fibrosis, and gut microbial composition. **Results:** Cel improved survival, left ventricular ejection fraction, fractional shortening, and cardiac output, while reducing LDH, LDH-1, CK, CK-MB, AST, *Anp*, and *Bnp*. It also decreased malondialdehyde levels, increased superoxide dismutase and glutathione peroxidase activities, alleviated mitochondrial swelling and cristae disruption, restored ATP production, and reduced myocardial fibrosis. In addition, Cel was associated with improved gut microbial diversity and composition, with *Bacteroides* and *Allobaculum* showing associations with host metabolic, antioxidant, and inflammatory indices. **Conclusions:** Cel attenuated DOX-induced cardiac injury by preserving mitochondrial integrity, improving redox and energy homeostasis, and limiting pathological remodeling. These cardioprotective effects were also accompanied by alterations in gut microbial composition, suggesting that Cel may represent a promising strategy for mitigating DOX-induced cardiotoxicity.

## 1. Introduction

Doxorubicin (DOX), a widely used anthracycline chemotherapeutic agent, is effective against multiple malignancies, but its clinical application is limited by dose-dependent cardiotoxicity [1], which may progress to irreversible myocardial injury, left ventricular dysfunction, chronic heart failure, and dilated cardiomyopathy [2]. In addition to direct cardiac injury, DOX disrupts intestinal microbial homeostasis and activates gut–organ axis responses [3], thereby contributing to gastrointestinal injury, cardiac remodeling, and systemic toxicity [4].

Mitochondrial dysfunction, oxidative stress, and disturbed energy metabolism are central mechanisms of DOX-induced cardiotoxicity [5]. DOX impairs mitochondrial oxidative phosphorylation, promotes excessive reactive oxygen species generation, and induces mitochondrial membrane lipid peroxidation, ultimately compromising mitochondrial integrity and bioenergetic function [6]. Thus, interventions that preserve mitochondrial homeostasis and suppress oxidative injury may be effective for mitigating DOX-associated cardiac damage.

Celastrol (Cel), a major bioactive triterpenoid quinone derived from *Tripterygium wilfordii* Hook. f. [7], has attracted increasing attention because of its multi-target pharmacological activities [8]. *Tripterygium wilfordii* and its related preparations have a long history of medicinal use, particularly in the management of chronic inflammatory and autoimmune disorders. Clinical studies have primarily evaluated *Tripterygium wilfordii* extracts in rheumatoid arthritis and other immune-mediated diseases, supporting their anti-inflammatory and immunosuppressive potential, although treatment is constrained by dose-dependent systemic toxicity [9]. Cel is considered one of the principal bioactive constituents contributing to the pharmacological properties of *Tripterygium wilfordii*. Unlike the crude botanical preparations, however, purified Cel has not yet acquired a well-established clinical indication and has been investigated mainly in preclinical models of inflammation, cancer, obesity, metabolic dysfunction, and tissue fibrosis [10,11,12]. Thus, the therapeutic development of Cel may be viewed as a natural-product-based drug-repositioning strategy that seeks to extend a pharmacologically well-characterized molecule beyond its traditional inflammatory and immune-related context.

Cardiovascular studies have begun to reveal that the anti-inflammatory, antioxidant, and anti-remodeling properties of Cel may also be relevant to cardiac disease. Cel has been reported to attenuate angiotensin II-induced cardiac remodeling through regulation of STAT3 and to reduce post-myocardial-infarction fibrosis by suppressing NLRP3 inflammasome activation [13,14]. Its potential utility has also been discussed in the context of heart failure with preserved ejection fraction [8]. Because DOX cardiotoxicity involves mitochondrial damage, oxidative stress, inflammatory activation, metabolic disruption, and pathological remodeling, the pleiotropic pharmacological profile of Cel provides a rationale for repositioning this compound as a candidate cardioprotective agent in the cardio-oncology setting.

Recent evidence further suggests that Cel can reshape gut microbial composition, stabilize the intestinal metabolic barrier, and preserve microbial homeostasis [7]. Given the emerging role of gut microbiota dysbiosis in DOX-induced systemic toxicity and cardiac remodeling, the Cel-associated microbial alterations may accompany its cardioprotective effects [15]. However, whether Cel can alleviate DOX-induced heart failure and intestinal dysbiosis remains unclear.

Therefore, this study investigated whether Cel could be therapeutically repositioned to protect against DOX-induced cardiotoxicity and prevent its progression toward heart failure. By integrating cardiac functional assessment, histopathology, mitochondrial ultrastructure, energy metabolism, oxidative stress indices, and gut microbiota profiling, we aimed to determine whether Cel exerts a previously underexplored cardioprotective function while also characterizing treatment-associated changes in gut microbial composition.

## 2. Results

### 2.1. Cel Intervention Improves Physiological Changes Induced by Dox-Induced Cardiotoxicity

**Figure 1:**
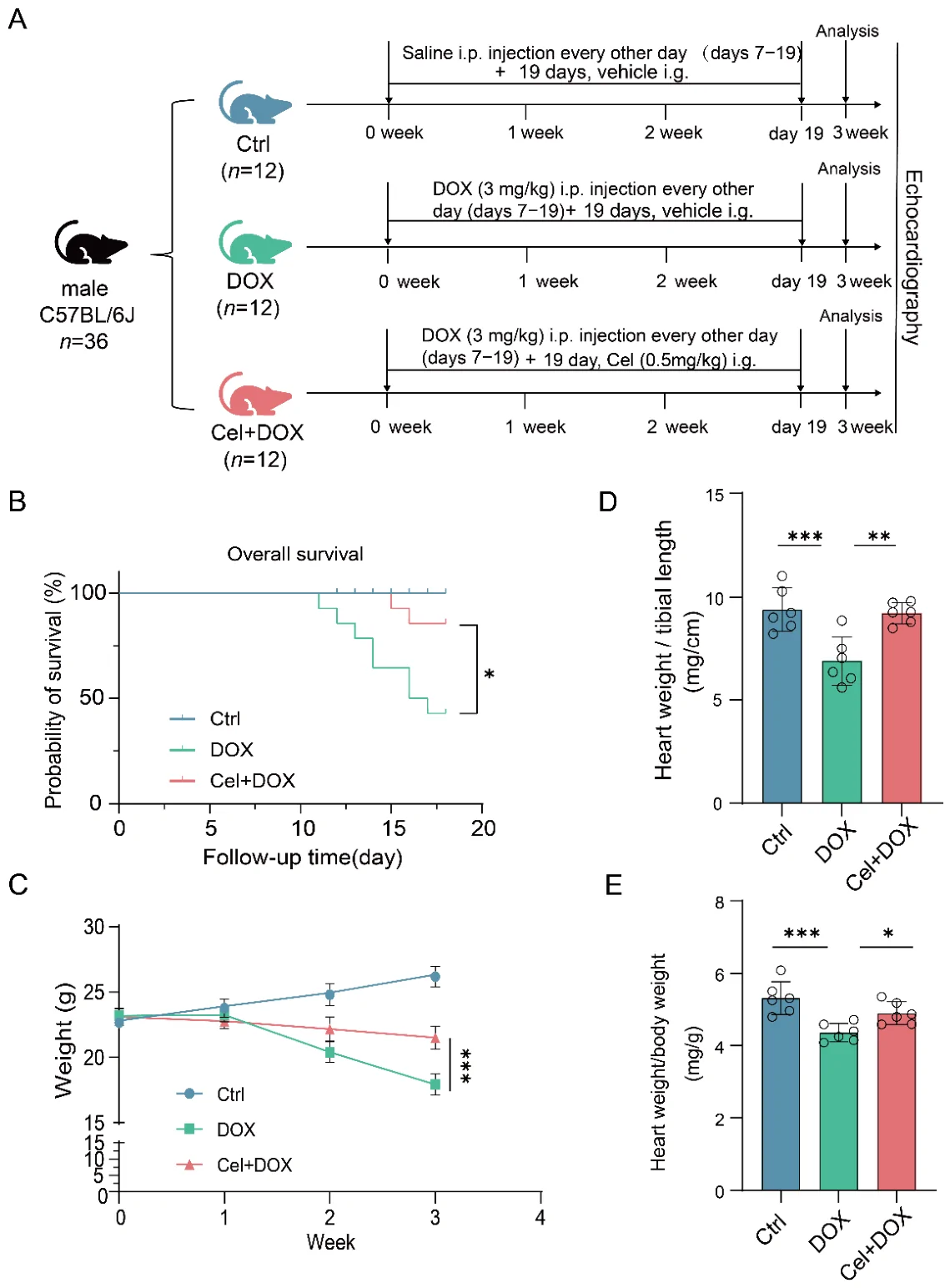
Cel intervention alleviates DOX-induced cardiotoxicity, improves overall health status, and reduces cardiac load in mice. (**A**) 57. BL/6J mice were randomly assigned to the Ctrl, DOX, and Cel + DOX groups (*n* = 12 per group). The DOX and Cel + DOX groups received intraperitoneal DOX injections, whereas the Ctrl group received saline. Mice in the Cel + DOX group were additionally administered Cel by oral gavage. Mice were euthanized on day 21, and samples were collected for further analyses. (**B**) Kaplan–Meier survival curve. (**C**) Body weight changes. (**D**,**E**) Heart weight-to-body weight ratio (HW/BW) (**D**) and heart weight-to-tibia length ratio (HW/TL) (**E**). Each data point represents an individual mouse. For B and C, *n* = 12 per group; for (**D**,**E**), *n* = 6 per group; and each data point represents an individual mouse. Significant differences between groups were indicated by *, where * indicates *p* < 0.5, ** indicates *p* < 0.01, *** indicates *p* < 0.001.

The experimental design is shown in Figure 1A. To evaluate whether either the absence of a separate healthy Cel-only group or the selected Cel dose could complicate interpretation of the therapeutic effect, we additionally summarized previously reported in vivo Cel dosing regimens, experimental grouping strategies, and safety-related observations (Table S1) [16,17,18,19,20,21,22,23,24,25,26,27,28,29,30,31,32,33,34,35,36,37,38,39,40,41,42]. Across the surveyed studies, Cel demonstrated pharmacological activity within an approximately 0.25–1 mg/kg range, and numerous disease-model efficacy studies evaluated Cel treatment by comparing model and Cel-treated model groups without including a separate healthy Cel-only group. This pattern indicates that omission of a healthy Cel-only group is a frequently used design in Cel efficacy studies when the principal objective is to assess intervention effects within a disease context rather than to characterize drug effects in healthy animals. Importantly, independent cardiac safety data from normal C57BL/6J mice showed that administration of 1 mg/kg/day for 2 weeks did not significantly alter heart weight, major cardiac structural parameters, or systolic function, whereas adverse cardiac changes were observed at higher repeated-dose exposures. Because the dose used in the present study was 0.5 mg/kg/day, it was below a reported dose at which no detectable cardiac structural or functional abnormalities were observed and substantially below doses associated with detectable cardiotoxicity. Taken together, the literature-based assessment supports two aspects of the present design: the three-group efficacy strategy is consistent with previous Cel intervention studies in disease models, and the selected 0.5 mg/kg/day dose is unlikely to produce independent cardiac structural or functional effects sufficient to explain the protective phenotype observed in the Cel + DOX group. These considerations diminish the possibility that the absence of a healthy Cel-only group materially confounded interpretation of the primary DOX versus Cel + DOX comparison.

After 21 days of treatment, DOX-treated mice exhibited marked physiological deterioration, including body weight loss, increased mortality, and altered cardiac morphometric indices. Cel intervention significantly alleviated DOX-induced body weight loss and improved survival compared with the DOX group (Figure 1B,C). The heart weight-to-body weight ratio (HW/BW) and heart weight-to-tibia length ratio (HW/TL) are critical indicators of heart function [43]. In addition, Cel partially restored the heart weight-to-body weight ratio (HW/BW) and heart weight-to-tibia length ratio (HW/TL), suggesting improvement in DOX-induced cardiac remodeling-associated changes (Figure 1D,E).

### 2.2. Cel Ameliorates Dox-Induced Pathological Cardiac Remodeling and Heart Failure Symptoms, with Reduced Left Ventricular Dilatation

**Figure 2:**
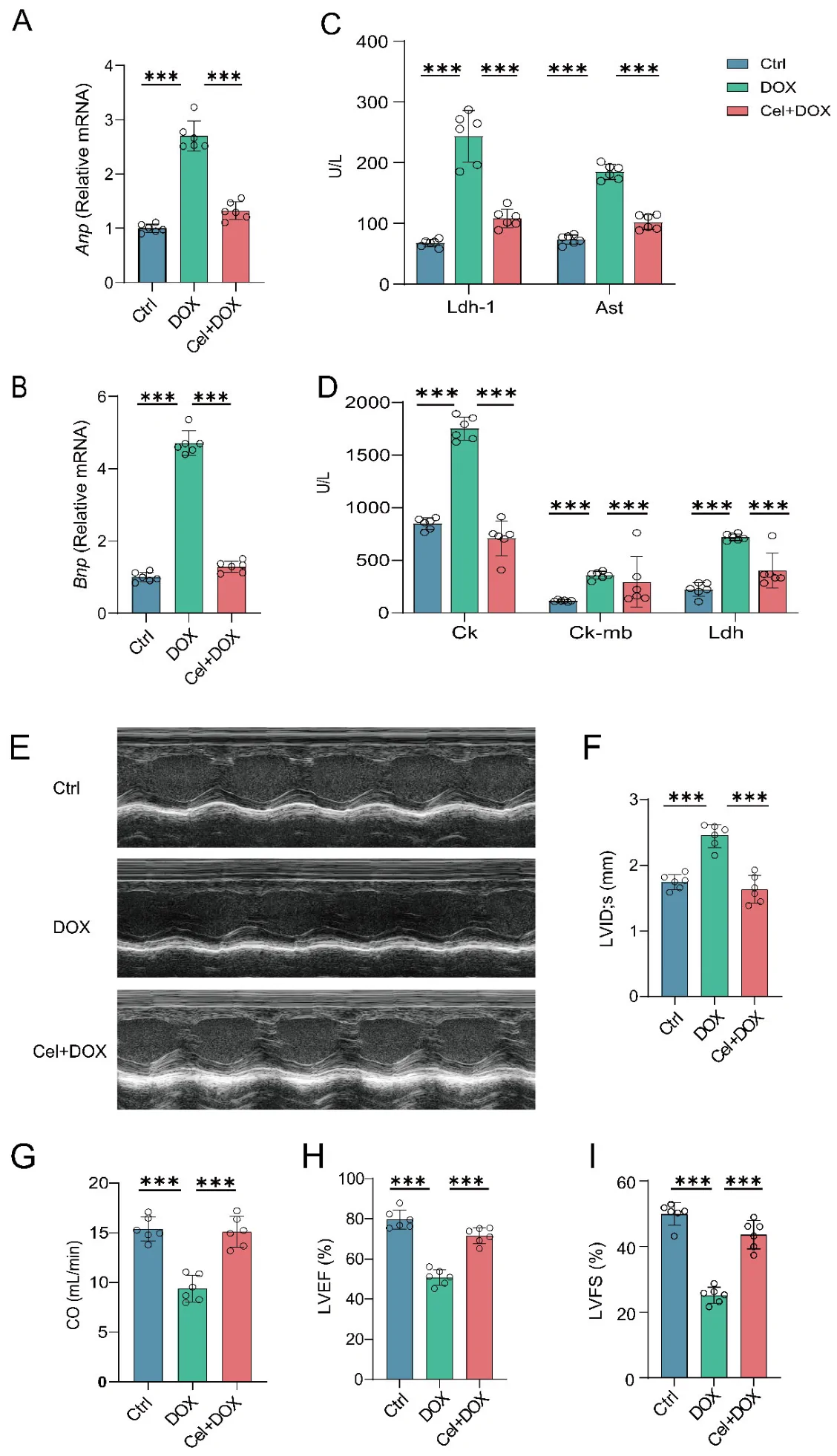
Cel ameliorates DOX-induced cardiac remodeling and left ventricular dilation. (**A**,**B**) mRNA expression of heart failure markers atrial natriuretic peptide (Anp, **A**) and brain natriuretic peptide (Bnp, **B**) in the heart. (**C**,**D**) Serum levels of myocardial injury biomarkers: lactate dehydrogenase isoenzyme (Ldh-1, **C**), aspartate aminotransferase (Ast, **C**), reatine kinase (Ck, **D**), creatine kinase-mb (Ck-mb, **D**), and lactate dehydrogenase (Ldh, **D**). (**E**) M-mode echocardiographic images showing normal left ventricular morphology in control mice, DOX-induced ventricular dilation, and the protective effects of Celin the Cel + DOX group. (**F**–**I**) Quantitative analysis of echocardiographic parameters: left ventricular internal dimension at systole (LVID;s, **F**), cardiac output (CO, **G**), left ventricular ejection fraction (LVEF, **H**), and left ventricular fractional shortening (LVFS, **I**). *n* = 6, and each data point represents an individual mouse. One-way analysis of variance (ANOVA) was used to compare the group differences. Significant differences between groups were indicated by *, where *** indicates *p* < 0.001.

The mRNA expression levels of the heart failure biomarkers atrial natriuretic peptide (*Anp*) and brain natriuretic peptide (*Bnp*) were significantly elevated in the DOX group compared with the normal control group [44]. However, Cel intervention markedly reduced the expression of both genes (Figure 2A,B) (*p* < 0.001), indicating its ability to effectively ameliorate DOX-induced cardiac dysfunction, alleviate heart failure, and suppress pathological cardiac remodeling [45]. In addition, myocardial enzymatic indicators are key clinical parameters for assessing the extent of myocardial injury and represent the most direct and reliable markers of cardiac damage [46]. Experimental results showed that levels of CK, CK-MB, LDH, LDH-1, and AST were all significantly elevated in the DOX group, indicating substantial myocardial cell damage (Figure 2C,D). In contrast, the levels of these myocardial injury markers were significantly lower in the Cel-treated group (*p* < 0.001). Since these biochemical indicators reflect the structural and functional integrity of the myocardium, the observed changes suggest that Cel mitigates DOX-induced myocardial injury and preserves cardiomyocyte integrity and function [47]. In summary, Cel effectively improved DOX-induced pathological cardiac remodeling and cardiac dysfunction by downregulating the expression of heart failure-related genes and reducing myocardial injury enzyme levels, thereby exerting a pronounced cardioprotective effect.

Echocardiographic assessment further confirmed the cardioprotective effect of Cel. M-mode echocardiography showed that LVIDs was significantly increased in the DOX group compared with the saline group (*p* < 0.001; Figure 2E,F), whereas Cel intervention markedly attenuated DOX-induced enlargement of the left ventricular internal diameter, indicating reduced ventricular dilatation. Consistent with the heart weight alterations shown in Figure 1, these findings suggest that Cel improves DOX-induced cardiac remodeling. Functional analysis further demonstrated that DOX markedly reduced CO, LVEF, and LVFS levels [48] (*p* < 0.001; Figure 2G–I), indicating impaired cardiac pumping capacity. In contrast, Cel treatment significantly restored these parameters compared with the DOX group (*p* < 0.001), suggesting improved ventricular systolic function. Collectively, Cel attenuates DOX-induced cardiac injury and dysfunction by suppressing heart failure-associated gene expression, reducing myocardial enzyme release, and improving ventricular structure and contractile performance, thereby exerting a robust cardioprotective effect.

### 2.3. Cel Attenuates Dox-Induced Myocardial Fibrosis and Improves Cardiomyocyte Atrophy

**Figure 3:**
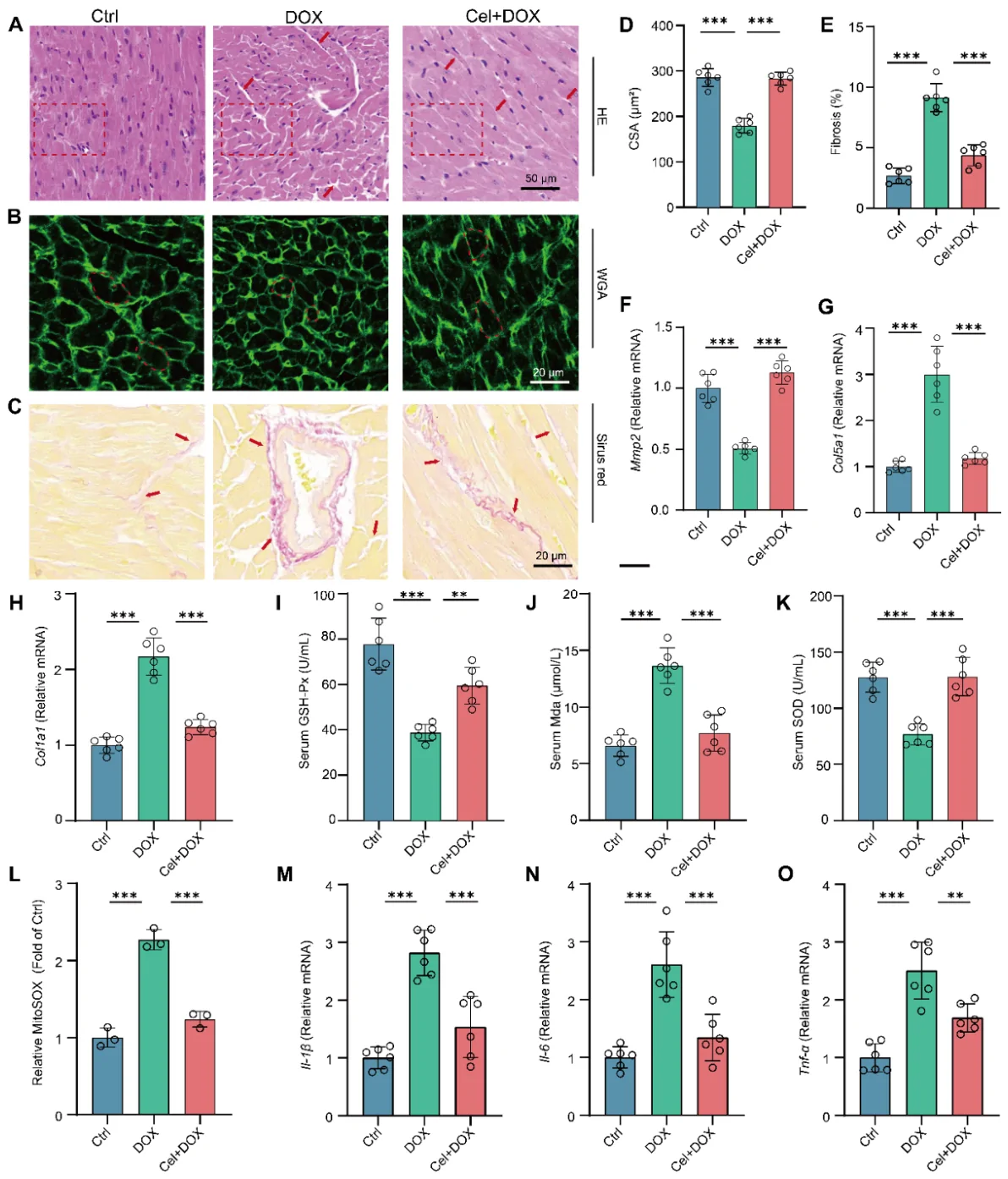
Cel attenuates DOX-induced myocardial structural injury, fibrosis, oxidative stress, and inflammation. (**A**) Representative hematoxylin and eosin (H&E) staining of myocardial tissue. The red dashed boxes indicate the representative regions selected for morphological observation, and red arrows indicate representative myocardial structural abnormalities, including disorganized myocardial fibers and widened interstitial spaces (scale bar = 50 μm). (**B**) Representative wheat germ agglutinin (WGA) staining. Red dashed outlines indicate representative cardiomyocyte profiles (scale bar = 20 μm). (**C**) Representative Sirius Red staining. Red arrows indicate areas of collagen deposition (scale bar = 20 μm). (**D**) Quantification of cardiomyocyte cross-sectional area (CSA). (**E**) Quantification of myocardial fibrosis. (**F**–**H**) Cardiac mRNA expression of Mmp2 (**F**), Col5a1 (**G**), and Col1a1 (**H**). (**I**–**K**) Serum GSH-Px activity (**I**), MDA levels (**J**), and SOD activity (**K**). (**L**) Relative mitochondrial superoxide levels assessed using MitoSOX. (**M**–**O**) Relative mRNA expression of the inflammatory markers Il-1β (**M**), Il-6 (**N**), and Tnf-α (**O**). Data are presented as mean ± SD. Each data point represents an individual biological replicate. Group differences were analyzed by one-way ANOVA followed by the appropriate multiple-comparisons test. Significant differences between groups were indicated by *, where ** indicates *p* < 0.01, *** indicates *p* < 0.001.

H&E staining revealed that DOX induced severe myocardial injury, characterized by cardiomyocyte swelling, fiber rupture, nuclear displacement, interstitial expansion, and disordered myocardial fiber arrangement (Figure 3A). Cel treatment substantially preserved myocardial morphology, as evidenced by intact cell membranes, tighter intercellular junctions, and reduced interstitial spacing. Sirius Red staining showed extensive perivascular and interstitial collagen deposition in the DOX group (Figure 3C,E), whereas Cel markedly attenuated myocardial fibrosis. Consistently, WGA staining demonstrated increased interstitial area, weakened WGA signals, and reduced cell size after DOX exposure, while Cel restored clear cell borders and compact cardiomyocyte alignment (Figure 3B,D). These findings indicate that Cel effectively mitigates DOX-induced myocardial structural damage, fibrosis, and cellular atrophy.

Consistent with the histological findings, DOX markedly altered the expression of genes associated with extracellular matrix remodeling. DOX treatment significantly downregulated matrix metallopeptidase 2 (*Mmp2)* expression (*p* < 0.001; Figure 3F), which is associated with aggravated left ventricular systolic dysfunction and cardiac deterioration [49]. Meanwhile, the fibrosis-associated collagen genes collagen type V alpha 1 chain (*Col5a1*) and collagen type I alpha 1 chain (*Col1a1*) were significantly upregulated, indicative of enhanced fibrotic remodeling in the myocardium (Figure 3G,H). Cel intervention reversed these alterations by increasing *Mmp2* expression and suppressing *Col5a1* and *Col1a1* levels, thereby attenuating DOX-induced myocardial fibrosis and structural remodeling.

DOX exposure was also associated with pronounced oxidative stress. Serum oxidative stress analysis further indicated that DOX reduced Sod and GSH-Px levels while markedly increasing MDA expression, reflecting impaired antioxidant defense and enhanced lipid peroxidation (Figure 3I–K). Given that SOD and GSH-Px are key enzymes responsible for superoxide and hydrogen peroxide metabolism [50,51], their reduction may promote ROS accumulation and oxidative stress. Cel treatment significantly restored SOD and GSH-Px levels and decreased MDA content (*p* < 0.001), demonstrating its antioxidant capacity.

Given the close association between oxidative stress and inflammatory activation in DOX-induced cardiac injury, we further examined the expression of inflammatory markers. DOX significantly increased the mRNA expression of interleukin 1 beta (*Il-1β*), interleukin 6 (*Il-6*), and tumor necrosis factor alpha (*Tnf-α*) compared with the control group (all *p* < 0.001; Figure 3M–O). Cel treatment markedly reduced *Il-1β* and *Il-6* expression (both *p* < 0.001 versus DOX) and significantly decreased *Tnf-α* expression (*p* < 0.01 versus DOX). These results indicate that Cel attenuates the DOX-induced myocardial inflammatory transcriptional response.

MitoSOX-based flow cytometric analysis further showed that DOX increased mitochondrial superoxide levels in H9c2 cardiomyoblasts, whereas Cel pretreatment significantly reduced this increase (Figure 3L).

Collectively, these findings demonstrate that Cel mitigates DOX-induced myocardial injury through coordinated attenuation of structural damage, cardiomyocyte atrophy, fibrotic remodeling, oxidative stress, and inflammatory activation.

### 2.4. Cel Preserves Mitochondrial Integrity and Supports Cardiac Energy Homeostasis

**Figure 4:**
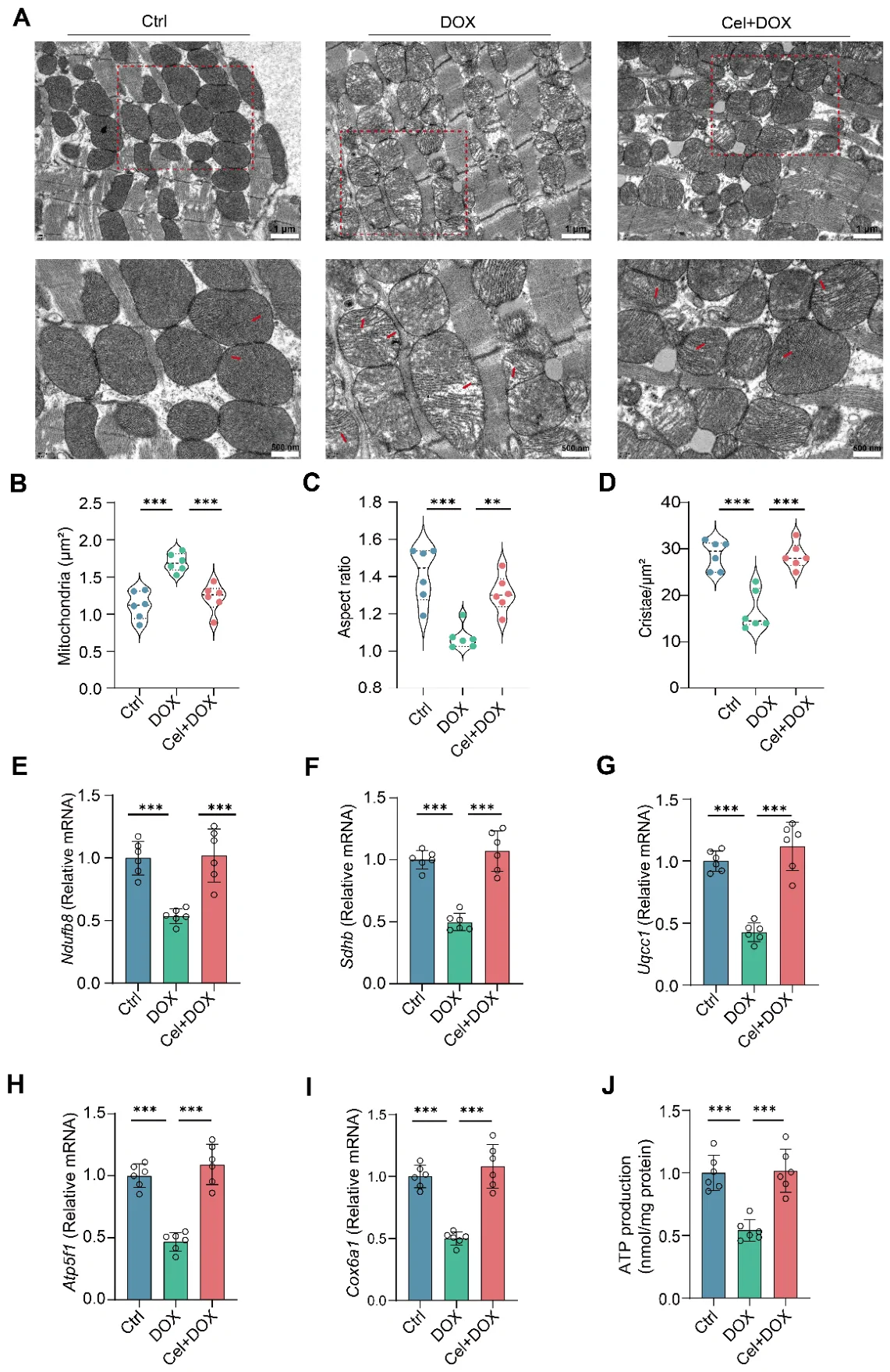
Cel alleviates DOX-induced mitochondrial damage, enhances energy production, and markedly reduces the impact of DOX toxicity on the ETC in cardiomyocytes. (**A**) TEM images of cardiomyocyte mitochondria (scale bars = 500 nm, 1000 nm). Red dashed boxes indicate the regions selected for higher-magnification imaging, and red arrows indicate mitochondrial cristae. Compared with the intact and densely arranged cristae in the Ctrl group, DOX treatment resulted in disrupted, fragmented, and sparsely arranged cristae, whereas Cel treatment markedly preserved cristae integrity and organization. (**B**–**D**) Quantitative analysis of mitochondrial parameters: mitochondrial area (**B**), aspect ratio (**C**), and cristae density (**D**). (**E**–**I**) mRNA expression of ETC complex-related genes; (**J**) ATP levels in cardiomyocytes. One-way analysis of variance (ANOVA) was used to compare the group differences. Significant differences between groups were indicated by *, where ** indicates *p* < 0.01 and *** indicates *p* < 0.001.

TEM analysis showed that DOX markedly disrupted mitochondrial ultrastructure in cardiomyocytes, as reflected by mitochondrial swelling, reduced cristae density, widened cristae spacing, and cristae fragmentation (Figure 4A). Quantitative analysis confirmed that DOX significantly increased mitochondrial cross-sectional area, reduced the aspect ratio, and decreased cristae density compared with the control group (*p* < 0.001; Figure 4B–D), indicating mitochondrial swelling and structural impairment. In contrast, Cel treatment significantly reduced mitochondrial swelling, restored mitochondrial aspect ratio, and increased cristae density compared with the DOX group (*p* < 0.001).

Consistently, DOX significantly reduced cardiac ATP content and downregulated the mRNA expression of electron transport chain-related genes, including NADH/ubiquinone oxidoreductase subunit B8 (*Ndufb8*), succinate dehydrogenase complex iron sulfur subunit B (*Sdhb*), ubiquinol-cytochrome c reductase complex assembly factor 1 (*Uqcc1*), cytochrome c oxidase subunit 6A1 (*Cox6a1*), and ATP synthase F1 subunit alpha (*Atp5f1a*) (*p* < 0.001; Figure 4E–J), indicating impaired mitochondrial energy metabolism. Cel intervention significantly restored ATP levels and the expression of these respiratory chain-related genes compared with the DOX group (*p* < 0.001). These findings support a protective effect of Cel on DOX-associated mitochondrial structural and bioenergetic alterations.

**Figure 5:**
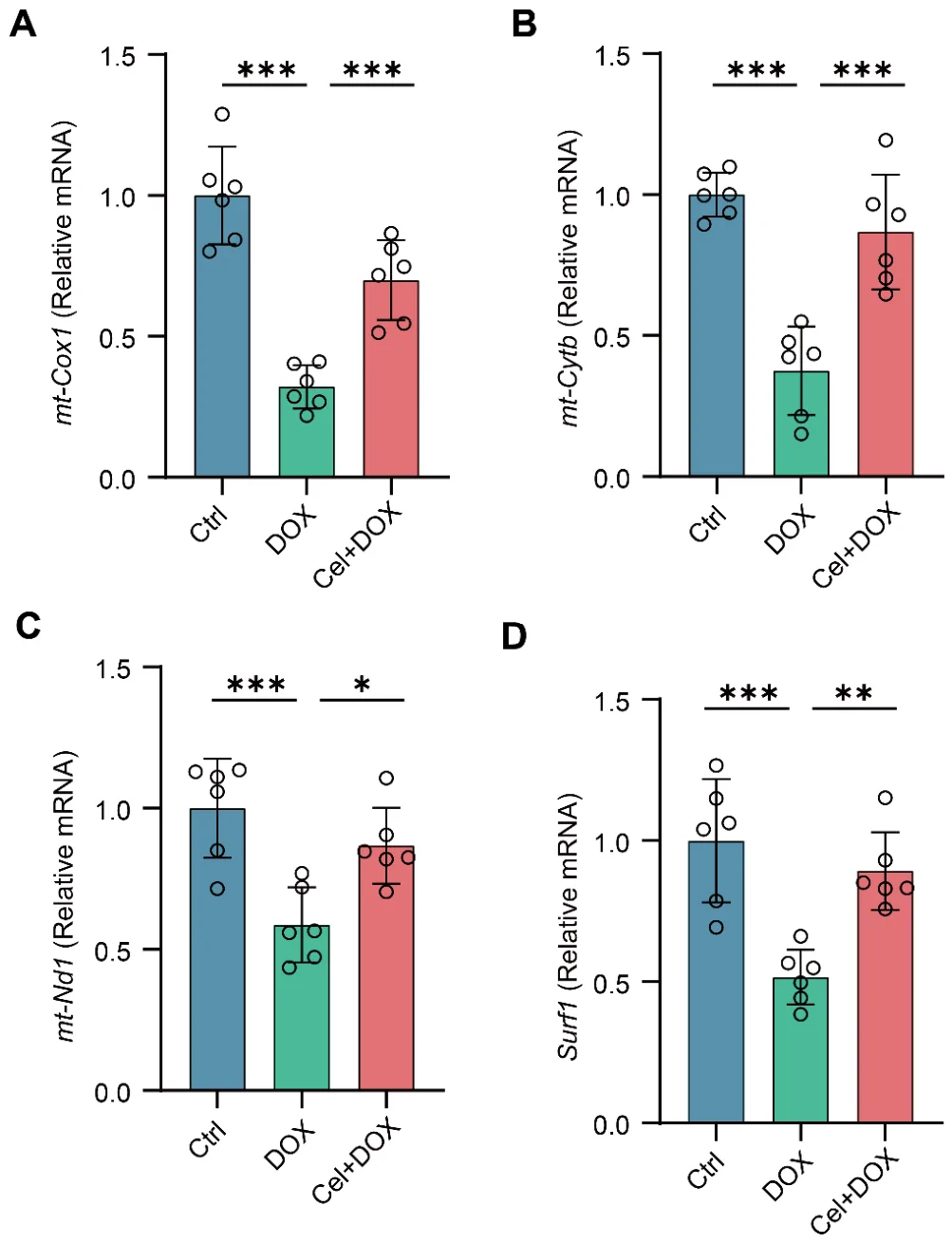
Cel partially reverses DOX-induced alterations in mitochondrial respiratory chain- and homeostasis-related gene expression. (**A**–**D**) Relative mRNA expression levels of mt-Cox1 (**A**), mt-Cytb (**B**), mt-Nd1 (**C**), Surf1 (**D**) in cardiac tissues from Ctrl, DOX, and Cel + DOX groups. Data are presented as mean ± SD (*n* = 6 per group), with each data point representing an individual mouse. Group differences were analyzed by one-way ANOVA followed by multiple-comparisons testing. * *p* < 0.05, ** *p* < 0.01, *** *p* < 0.001.

Additional analysis of mitochondrial respiratory-chain and homeostasis-related genes showed that DOX significantly reduced *mt-Cox1*, *mt-Cytb*, *mt-Nd1*, and *Surf1* expression compared with the control group (all *p* < 0.001; Figure 5A–D). Cel treatment significantly increased these transcripts relative to the DOX group (*mt-Cox1* and *mt-Cytb*, *p* < 0.001; *mt-Nd1*, *p* < 0.05; *Surf1*, *p* < 0.01). These findings indicate that Cel partially reverses DOX-induced transcriptional disturbances in genes associated with the mitochondrial respiratory chain and mitochondrial homeostasis.

### 2.5. Cel Treatment Is Associated with Partial Recovery of Gut Microbial Diversity and Changes in Community Composition

**Figure 6:**
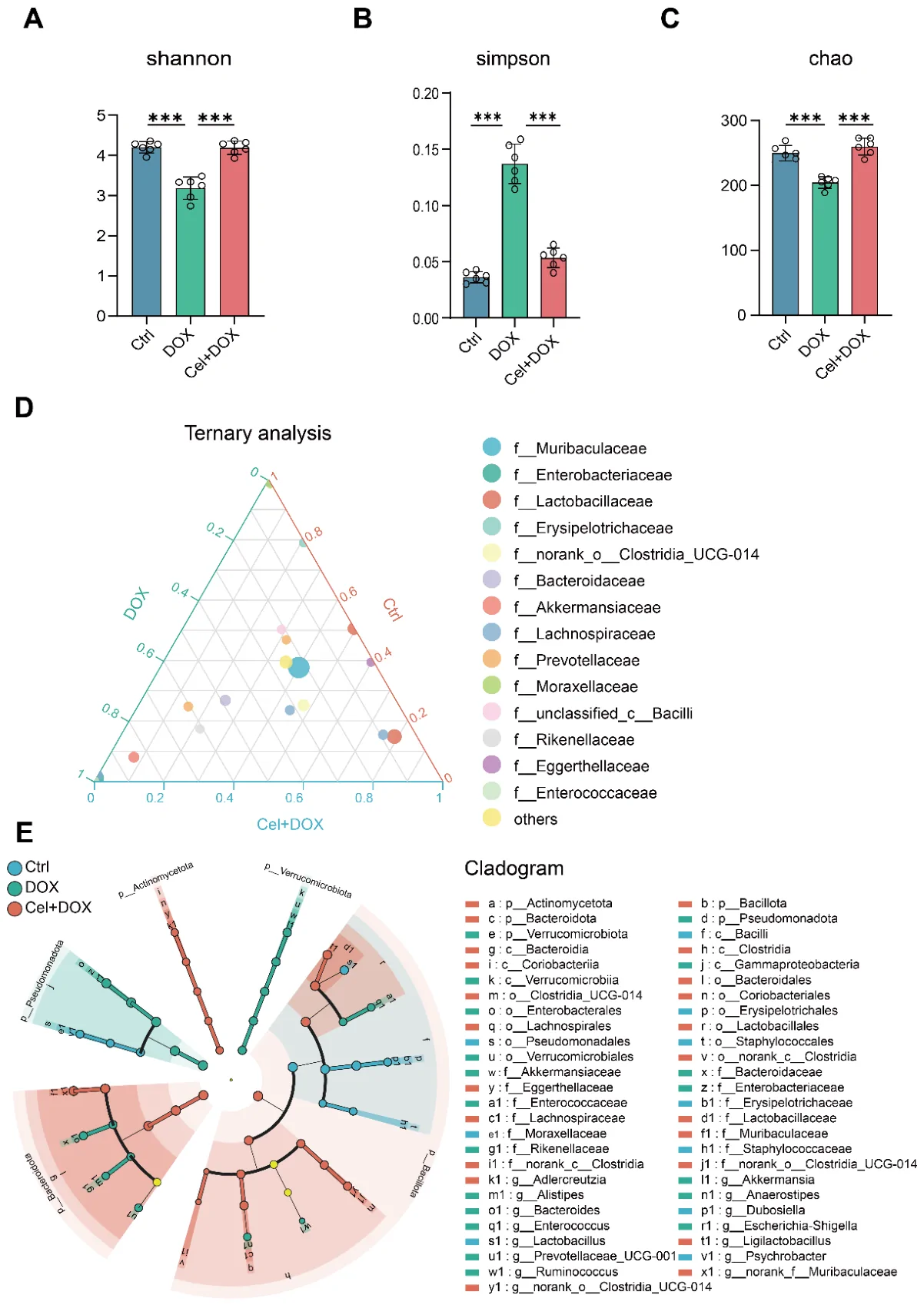
Cel treatment is associated with changes in gut microbial diversity and composition. (**A**–**C**) α-diversity analysis: the Shannon index (**A**), the Simpson index (**B**), the Chao index (**C**), and group differences were analyzed using the Kruskal–Wallis test, followed by Dunn’s multiple-comparisons test; (**D**) Ternary plot analysis; (**E**) Phylogenetic cladogram analysis (Blue, green, and coral-red nodes indicate taxa enriched in the Ctrl, DOX, and Cel + DOX groups, respectively, whereas yellow nodes represent taxa that were not significantly enriched in any group). *n* = 6, and each data point represents an individual mouse group. Group differences were analyzed using the Kruskal–Wallis test followed by Dunn’s multiple-comparisons test. Data are presented as median with interquartile range. *** *p* < 0.001.

16S rRNA gene sequencing was performed to evaluate the effect of Cel on DOX-induced gut microbiota dysbiosis. DOX treatment significantly reduced gut microbial α-diversity, as indicated by decreased Shannon and Chao indices compared with the control group (*p* < 0.001; Figure 6A,C), whereas Cel treatment significantly restored both indices (*p* < 0.001). The Simpson index was also significantly altered by DOX and reversed by Cel intervention (*p* < 0.001; Figure 6B), further suggesting restoration of microbial diversity.

Ternary plot analysis revealed distinct microbial distribution patterns among the three groups (Figure 6D). DOX increased dysbiosis- and inflammation-associated taxa, particularly Enterobacteriaceae, whereas the control group was enriched in commensal taxa, including *Muribaculaceae*, *Lachnospiraceae*, and *Akkermansiaceae*. Cel shifted the microbial profile toward that of the control group. Phylogenetic analysis further showed enrichment of Actinobacteria and Enterobacteriaceae in the DOX group, while Cel increased potentially beneficial taxa, including *Akkermansia*, *Muribaculaceae*, and *Lachnospiraceae* (Figure 6E). These results indicate that Cel treatment was associated with partial shifts in microbial diversity and community composition toward the control profile by restoring microbial diversity and promoting a more favorable microbial community structure.

### 2.6. Associations Between Cel-Responsive Microbial Taxa and Host Indices

**Figure 7:**
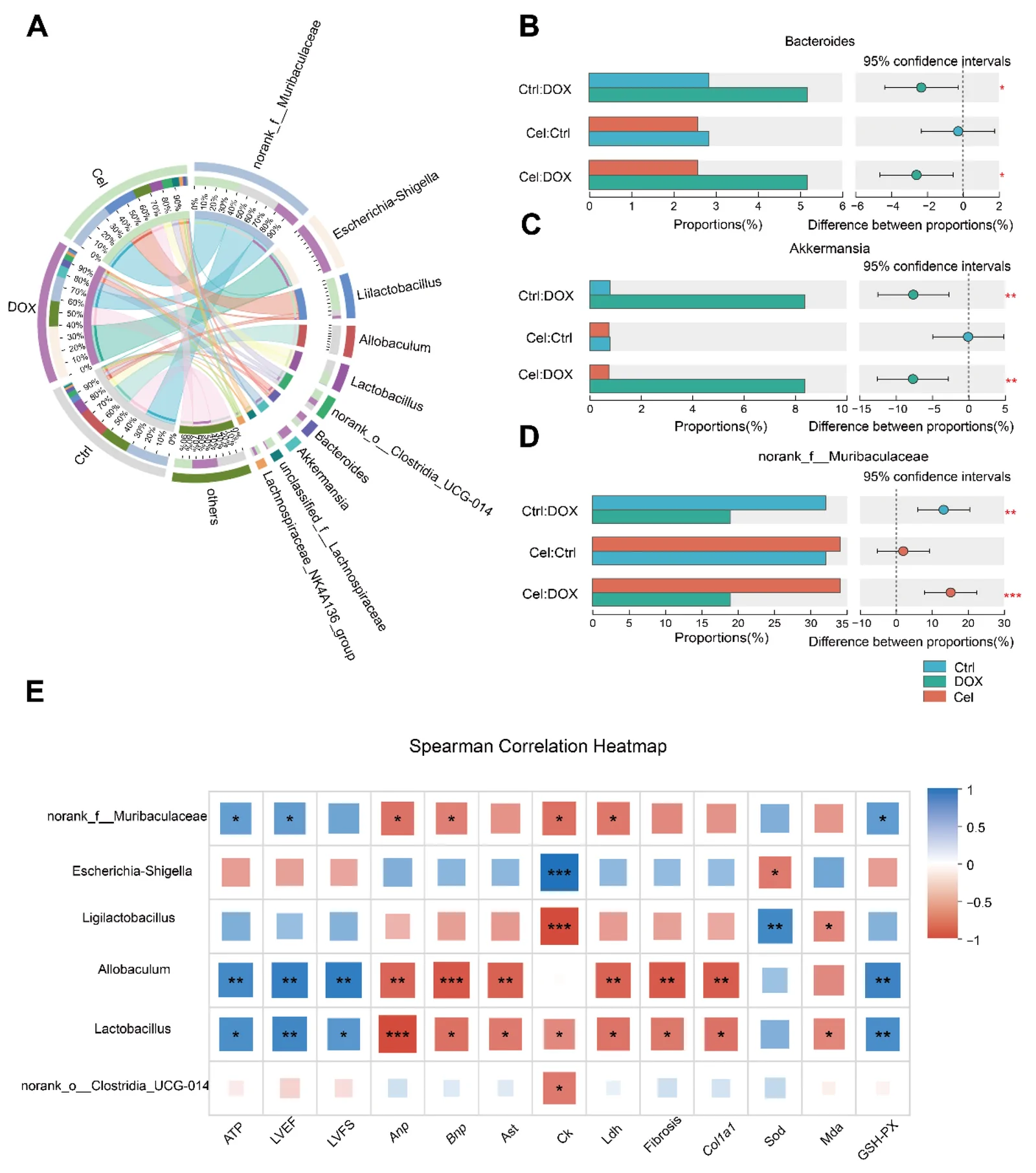
Cel modulates key microbial taxa and their association with cardiac injury parameters. (**A**) Genus-level community structure (circos plot), showing the relative abundance of the top genera in each group; (**B**–**D**) Differential abundance analysis of key genera; (**E**) Spearman correlation heatmap. *n* = 6, and each data point represents an individual mouse group. Group differences were analyzed using the Kruskal–Wallis test, followed by Dunn’s multiple-comparisons test. Data are presented as median with interquartile range. * *p* < 0.05, ** *p* < 0.01, *** *p* < 0.001.

Taxonomic profiling and Spearman correlation analyses were performed to further characterize Cel-responsive microbial changes. Taxonomic analysis showed distinct microbial community structures among the three groups (Figure 7A). DOX enriched opportunistic pathogenic taxa, particularly *Escherichia–Shigella* and Enterobacteriaceae, whereas the control group was dominated by commensal taxa such as *Muribaculaceae* and *Lachnospiraceae*. Cel intervention shifted the microbial profile toward that of the control group.

Relative abundance analysis showed that DOX significantly reduced *Bacteroides*, *Akkermansia*, and *norank_f__Muribaculaceae*, whereas the relative abundances of these taxa were higher in the Cel + DOX group than in the DOX group [52] (Figure 7B–D). Notably, *norank_f__Muribaculaceae* abundance was higher in the Cel-treated group than in the control group, suggesting that this taxon may be particularly responsive to Cel intervention.

Spearman correlation analysis showed that *norank_f__Muribaculaceae* and *Lactobacillus* were positively correlated with ATP levels and SOD activity but negatively correlated with AST, CK, and LDH levels (Figure 7E). In contrast, *Escherichia–Shigella* was positively correlated with LDH and negatively correlated with SOD activity [53], while *Allobaculum* was positively associated with AST, CK, and LDH but negatively associated with ATP levels and LVEF. These findings suggest that Cel-responsive microbial taxa are associated with host energy metabolism, oxidative stress, myocardial injury, and cardiac function.

## 3. Discussion

This study demonstrates that Cel protects against DOX-induced cardiomyopathy in male C57BL/6J mice. Cel improved cardiac function, reduced myocardial injury and fibrosis, suppressed oxidative stress, and preserved mitochondrial structure and energy metabolism. Cel treatment was also accompanied by changes in gut microbial diversity and composition. These findings support antioxidant, mitochondrial, and anti-remodeling effects of Cel, whereas the biological contribution of the associated microbial alterations remains to be established.

Mitochondrial dysfunction and oxidative stress are central mechanisms of DOX-induced cardiotoxicity [54,55]. In this study, DOX caused mitochondrial swelling, cristae disruption, decreased ATP production, and downregulation of electron transport chain-related genes, indicating impaired mitochondrial bioenergetic function [56].

Cel preserved mitochondrial ultrastructure, restored ATP levels, and partially reversed DOX-induced alterations in respiratory chain-related gene expression. In particular, the reductions in *mt-Cox1*, *mt-Cytb*, *mt-Nd1*, and *Surf1* following DOX exposure were significantly alleviated by Cel treatment, further supporting its protective effect against DOX-induced mitochondrial alterations [55]. Nevertheless, mitochondrial oxygen consumption and respiratory complex activities were not directly assessed in the present study; therefore, the ATP and gene-expression data should be interpreted as evidence of improved mitochondrial bioenergetic homeostasis rather than direct restoration of electron transport chain respiratory activity.

In parallel, MitoSOX-based flow cytometric analysis showed that DOX markedly increased mitochondrial superoxide levels, whereas Cel treatment significantly attenuated this increase. Consistent with these findings, DOX reduced SOD and GSH-Px activities and increased MDA levels, while Cel significantly reversed these changes. Together, the MitoSOX results and biochemical oxidative stress indices provide complementary evidence that Cel alleviates DOX-induced mitochondrial oxidative stress [32]. This antioxidant effect may contribute, at least in part, to the cardioprotective action of Cel and is consistent with previous reports describing its antioxidant properties.

Inflammatory activation accompanied the oxidative and mitochondrial disturbances induced by DOX, whereas Cel reduced the induction of *Il-1β*, *Il-6*, and *Tnf-α*. Cel also partially restored the expression of several genes involved in mitochondrial respiratory-chain function and assembly, including *mt-Cox1*, *mt-Cytb*, *mt-Nd1*, and *Surf1*. Together with the structural, ATP, and mitochondrial ROS data, these findings suggest that Cel mitigates a broader state of inflammatory and mitochondrial stress induced by DOX. Nevertheless, direct restoration of mitochondrial respiratory function cannot be inferred in the absence of measurements of oxygen consumption or respiratory complex activity.

Cardiotoxicity can be comprehensively evaluated in terms of electrophysiological abnormalities, contractile dysfunction, and structural remodeling [57,58].The protective effects of Cel were further confirmed by functional, biochemical, and histological evidence. Cel improved DOX-induced reductions in LVEF, LVFS, and cardiac output; reduced myocardial enzyme release and heart failure-related gene expression; and alleviated myocardial structural disruption, collagen deposition, and cardiomyocyte atrophy. The downregulation of *Col1a1* and *Col5a1* and restoration of *Mmp2* expression further suggest that Cel attenuates extracellular matrix remodeling and pathological cardiac fibrosis [13,14].

Cel treatment was also accompanied by alterations in gut microbial composition. DOX reduced α-diversity and increased opportunistic pathogenic taxa, including *Escherichia–Shigella* and Enterobacteriaceae [52], whereas Cel partially restored microbial diversity and enriched beneficial commensal taxa such as *Akkermansia*, *Bacteroides*, *Muribaculaceae*, and *Lachnospiraceae* [59]. These taxa are associated with intestinal barrier maintenance, short-chain fatty acid (SCFA) production [60], and metabolic homeostasis. Correlation analysis further linked Cel-responsive taxa with ATP levels, SOD activity, myocardial injury markers, and cardiac function. Although these associations do not establish causality, they suggest a potential gut–heart axis contribution to Cel-mediated cardioprotection [3].

The absence of a separate healthy Cel-only group was carefully considered in relation to the primary objective of the present study, the design of previous Cel efficacy studies, and the available normal-animal safety evidence. The primary aim of this study was to determine whether Cel attenuates DOX-induced cardiac injury. Therefore, the key therapeutic comparison was between the DOX and Cel + DOX groups, whereas a healthy Cel-only group would primarily address a different question, namely the independent physiological or toxicological effects of Cel under normal conditions. In this context, the lack of a Cel-only group does not preclude assessment of the principal efficacy question addressed in the present study.

This efficacy-oriented grouping strategy is also consistent with numerous previously published in vivo Cel intervention studies in disease models, in which therapeutic effects were evaluated by comparing disease-control and Cel-treated disease groups without including a separate healthy Cel-only group (Table S1). Thus, omission of a healthy Cel-only group is not uncommon in preclinical Cel efficacy studies when the primary objective is therapeutic intervention rather than characterization of drug effects in healthy animals. Importantly, this literature precedent was not used as the sole justification for the present design; rather, it was considered together with independent published evidence regarding the safety and tolerability of the selected Cel dose.

Because Cel has a relatively narrow therapeutic window, we specifically evaluated whether the 0.5 mg/kg/day dose used in the present study could itself influence cardiac structure or function and thereby confound interpretation of the observed protective effects. Our literature-based assessment showed that Cel has demonstrated pharmacological activity at approximately 0.25–1 mg/kg in multiple animal models, including repeated administration of 0.5 mg/kg for several weeks (Table S1). Importantly, available cardiac safety data in normal C57BL/6J mice showed no significant alterations in heart weight, major cardiac structural parameters, or systolic function following administration of 1 mg/kg/day for 2 weeks, whereas adverse cardiac changes were reported at higher repeated-dose exposures. Thus, the 0.5 mg/kg/day regimen used here was below a reported dose at which no detectable cardiac structural or functional abnormalities were observed and substantially below doses associated with detectable cardiotoxicity.

Taken together, three considerations support the feasibility of the present three-group design. First, the study was designed to address a therapeutic efficacy question rather than to characterize the independent pharmacological or toxicological effects of Cel in healthy animals. Second, similar grouping strategies have been frequently used in previous Cel efficacy studies conducted in disease models. Third, independent normal-animal cardiac safety data indicate that the selected 0.5 mg/kg/day dose has a low likelihood of producing cardiac structural or functional effects sufficient to account for the protective phenotype observed in the Cel + DOX group. Therefore, although the present design does not directly characterize the independent effects of Cel under physiological conditions, the absence of a separate healthy Cel-only group is unlikely to materially compromise interpretation of the primary comparison between the DOX and Cel + DOX groups.

Several limitations should be acknowledged. First, only male mice and a single Cel dose were evaluated, and therefore sex-dependent responses and formal dose–response relationships remain to be determined. Because the present study was designed specifically to evaluate the therapeutic effect of Cel in DOX-induced cardiac injury, it did not directly characterize the independent physiological effects of Cel in healthy animals. However, this question is distinct from the primary efficacy comparison between the DOX and Cel + DOX groups, and the selected 0.5 mg/kg/day dose was supported by published normal-animal tolerability and cardiac safety data. Future studies specifically focused on defining the physiological or toxicological profile of Cel may include healthy Cel-treated controls and broader dose-ranging designs [61]. Second, because terminal analyses in the DOX group were performed in the surviving mice following substantial mortality, potential survivor-selection bias cannot be excluded. Although mitochondrial ultrastructure, ATP levels, and mitochondrial-related gene expression were evaluated, mitochondrial respiration and apoptosis-related pathways were not directly assessed. Third, the gut microbiota findings are associative and do not establish a causal role of microbial alterations in Cel-mediated cardioprotection; microbiota-derived metabolites, including SCFAs, were also not directly measured. Future studies using metabolomic analyses and microbiota-manipulation approaches will be needed to clarify these relationships. Finally, longer follow-up periods and additional experimental models are required to further evaluate the long-term efficacy and translational relevance of Cel.

An important implication of the present study is the potential therapeutic repositioning of Cel from its traditional inflammatory and immune-related pharmacological context to the prevention of chemotherapy-associated cardiac injury. Preparations derived from *Tripterygium wilfordii* have historically been investigated or used for rheumatoid arthritis and other chronic immune-mediated disorders, whereas purified Cel has been studied predominantly for its anti-inflammatory, antitumor, and metabolic regulatory activities [9,62]. Although previous cardiovascular studies have demonstrated beneficial effects of Cel in angiotensin II-induced cardiac remodeling, myocardial infarction-associated fibrosis, and experimental conditions relevant to heart failure [13], its role in DOX-induced heart failure has remained insufficiently defined. The present findings extend the pharmacological spectrum of Cel to an anthracycline cardiotoxicity setting and identify a previously underexplored function of this compound in limiting the progression from chemotherapy-induced myocardial injury to cardiac dysfunction and heart failure.

## 4. Materials and Methods

### 4.1. Chemicals and Reagents

Doxorubicin (DOX; HPLC ≥ 99%; D8740) was purchased from Solarbio Life Science Co., Ltd. (Beijing, China). Celastrol (Cel; purity 99.64%; HY13067) was obtained from MedChemExpress Co., Ltd. (Monmouth Junction, NJ, USA).

### 4.2. Animals and Experimental Design

Male C57BL/6 mice (6 weeks old; 20–25 g) were sourced from Vital River Laboratory Animal Technology Co., Ltd. (Beijing, China) and maintained in a specific pathogen-free facility under controlled environmental conditions, with a 12 h light–dark cycle and food and water available ad libitum. After one week of acclimatization, mice were randomly assigned to the Control, DOX, and Cel + DOX groups (*n* = 12 per group). The experimental grouping was designed according to the primary objective of this study, namely to determine whether Cel treatment attenuates DOX-induced cardiotoxicity. Accordingly, the comparison between the Control and DOX groups was used to establish DOX-induced cardiac injury, whereas the comparison between the DOX and Cel + DOX groups directly addressed the primary therapeutic question of whether Cel modifies the pathological phenotype induced by DOX. A separate healthy Cel-only group was not included because the present study was designed as an efficacy study in an established DOX-induced disease context rather than as a study intended to characterize the independent physiological or toxicological effects of Cel under normal conditions. In this efficacy-oriented design, a healthy Cel-only group would answer a distinct question—whether Cel alone alters physiological cardiac phenotypes in otherwise healthy animals—whereas the primary treatment-associated difference is evaluated directly through the comparison between the DOX and Cel + DOX groups. This grouping strategy is also consistent with numerous previously published in vivo Cel intervention studies in disease models, in which therapeutic efficacy was assessed by comparing model and Cel-treated model groups without a separate healthy Cel-only group (Table S1). Importantly, this literature precedent was not used as the sole justification for the grouping strategy; the omission of a Cel-only group was considered together with independent published safety and tolerability evidence for the selected Cel dose.

Cel was initially dissolved in DMSO (Solarbio Life Science Co., Ltd., Beijing, China) and subsequently diluted with sterile PBS (Solarbio Life Science Co., Ltd., Beijing, China) to the required concentration. The corresponding vehicle consisted of sterile PBS containing the same final concentration of DMSO (2% [*v*/*v*] for the 0.5 mg/kg cel treatment) and was administered at an equivalent volume [63]. Mice in the Cel-prevention group received cel (0.5 mg/kg) once daily by oral gavage from day 1, with treatment initiated 6 days before DOX administration and continued throughout the experimental period. Cel was administered orally at 0.5 mg/kg/day. This dose was selected on the basis of a literature-based assessment of previously reported in vivo Cel dosing regimens, pharmacological efficacy, experimental grouping strategies, and safety/tolerability observations (Table S1). Across multiple animal models, Cel has demonstrated pharmacological activity within an approximately 0.25–1 mg/kg dose range, including repeated administration of 0.5 mg/kg for several weeks. Importantly, available data from normal C57BL/6J mice showed no significant alterations in heart weight, major cardiac structural parameters, or systolic function following administration of 1 mg/kg/day for 2 weeks, whereas higher repeated-dose exposures of 2 mg/kg/day and 4 mg/kg every other day were associated with detectable adverse cardiac changes. Therefore, the 0.5 mg/kg/day dose used in the present study was below the reported exposure at which no detectable cardiac structural or functional abnormalities were observed and substantially below exposure levels associated with detectable cardiotoxicity. On this basis, 0.5 mg/kg/day was selected as a pharmacologically active but conservative exposure. The combination of the efficacy-oriented study design and the available normal-animal cardiac safety evidence minimizes the likelihood that Cel itself would produce cardiac structural or functional alterations sufficient to materially confound the primary comparison between the DOX and Cel + DOX groups [36,64,65].

Mice in the DOX model group received an equivalent volume of vehicle by oral gavage from days 1 to 19 and were administered DOX intraperitoneally (3 mg/kg in saline) every other day from days 7 to 19, with a total of seven injections. Mice in the Cel-prevention group received the same DOX regimen from days 7 to 19 while daily cel administration was maintained. Control mice received an equivalent volume of vehicle by oral gavage from days 1 to 19 and intraperitoneal saline from days 7 to 19 according to the same injection schedule used for the DOX-treated groups [66,67]. The final DOX injection was administered on day 19, and echocardiography was performed on day 21. Mice were then anesthetized with 2% isoflurane (Solarbio Life Science Co., Ltd., Beijing, China) and euthanized by cervical dislocation. Initially, 12 mice were assigned to each group, and all animals were included in the survival analysis. Approximately 50% mortality occurred in the DOX group during the experimental period, leaving six mice alive at the endpoint. All six surviving DOX-treated mice were therefore included in the subsequent terminal analyses. For endpoint analyses reported as *n* = 6, six mice were randomly selected from the Control and Cel + DOX groups to maintain a consistent sample size across groups.

### 4.3. Ethical Approval

All animal procedures were approved by the Animal Ethics Committee of China Agricultural University (Beijing, China; approval no. AW82805202-5-01; 28 August 2025) and performed in accordance with the Guidelines for the Care and Use of Laboratory Animals.

### 4.4. Echocardiography

Transthoracic echocardiography was performed in mice anesthetized with 1.5% isoflurane using a Vevo 3100 high-resolution microimaging system (FUJIFILM VisualSonics, Toronto, ON, Canada). Long-axis and short-axis cardiac views were acquired, and left ventricular (LV) function was assessed at a frame rate of 233 Hz. M-mode images at the papillary muscle level were used to measure LV end-diastolic diameter (LVEDD) and LV end-systolic diameter (LVESD), with values averaged from 3 to 5 consecutive cardiac cycles. Left ventricular ejection fraction (LVEF) was calculated as follows: $LVEF\left(\%\right)=\frac{\left(LVIDd^{3}-LVIDs^{3}\right)}{LVIDd^{3}}$; here, LVIDd and LVIDs represent LV internal diameters at diastole and systole, respectively. All echocardiographic analyses were performed in a blinded manner.

### 4.5. Data Recording and Biological Material Collection

Body weight and tibial length were recorded, and 2–3 fresh fecal pellets were collected for gut microbiota analysis. Blood samples were obtained by retro-orbital collection for serum biochemical assays. After euthanasia, hearts were perfused with 0.1 M PBS (pH 7.4), excised, weighed, and sectioned at the maximal transverse plane. Heart tissues were allocated for histological staining, RNA extraction, ATP measurement, and transmission electron microscopy.

### 4.6. Histological Analysis

Heart tissues were fixed overnight in 4% paraformaldehyde (pH 7.4), embedded in paraffin, and sectioned at 5 μm thickness. Hematoxylin and eosin staining (H&E; G1120, Solarbio, Beijing, China) was performed for routine histological assessment. Sirius Red staining (G1472, Solarbio, Beijing, China) was used to evaluate myocardial collagen deposition and fibrosis, whereas wheat germ agglutinin staining (WGA; W11261, Thermo Fisher Scientific, Waltham, MA, USA) was performed to assess cardiomyocyte cross-sectional area. Stained sections were imaged using a Zeiss Axioplan 2 optical microscope (Zeiss, Oberkochen, Germany). For each mouse, three adjacent sections were analyzed, and quantitative measurements were performed using ImageJ software (version 1.52, National Institutes of Health, USA).

### 4.7. Biochemical Marker Analysis

Blood samples were allowed to clot at room temperature for 2 h and centrifuged at 3000 rpm for 15 min at 2–8 °C to obtain serum. Serum levels of aspartate aminotransferase (AST), creatine kinase (CK), lactate dehydrogenase (LDH), LDH isoenzyme 1 (LDH-1), and creatine kinase-MB (CK-MB) were measured using commercial assay kits according to the manufacturers’ instructions. AST, CK, and LDH were detected using kits from Rayto Life and Analytical Sciences Co., Ltd. (S03040, S03024, and S03034; Shenzhen, China), whereas LDH-1 and CK-MB were measured using kits from Changchun Huili Biotech Co., Ltd. (C058-e and C060; Changchun, China). Enzymatic activities were determined using an automated biochemical analyzer (Chemray 800, Rayto Life and Analytical Sciences Co., Ltd., Shenzhen, China) by the rate method at 340 nm with a secondary wavelength of 405 nm.

Serum oxidative stress markers, including malondialdehyde (MDA), superoxide dismutase (SOD), and glutathione peroxidase (GSH-Px), were measured using commercial kits from Nanjing Jiancheng Bioengineering Institute (MDA, A003-1; SOD, A001-3; GSH-Px, A005-1; Nanjing, China) according to the manufacturer’s protocols. Absorbance was measured using a microplate reader (W11261, Thermo Fisher Scientific, Waltham, MA, USA), and marker levels were calculated according to the kit instructions.

### 4.8. Transmission Electron Microscopy (Tem)

Fresh heart tissues were fixed in 0.1 mol/L sodium cacodylate buffer (pH 7.4) containing 2% glutaraldehyde and 2.5% paraformaldehyde for 1 h at room temperature, followed by overnight fixation at 4 °C. Samples were dehydrated through a graded ethanol series, embedded in Epon 812 epoxy resin, and polymerized at 60 °C for 24–48 h. Ultrathin sections (80–90 nm) were prepared using a Leica Ultracut E ultramicrotome (Leica Microsystems GmbH, Wetzlar, Germany)), stained with uranyl acetate and lead citrate, and examined using a JEOL 1400 transmission electron microscope (JEOL Ltd., Akishima, Tokyo, Japan). Images were captured with a Gatan Orius SC1000 CCD camera (Gatan, Inc., Pleasanton, CA, USA) and quantitatively analyzed using ImageJ software (version 1.54f, National Institutes of Health, Bethesda, MD, USA). Mitochondrial area was defined as the cross-sectional area of individual mitochondria, aspect ratio as the ratio of the major to minor axis, and cristae density as the number of cristae per mitochondrial area (cristae/μm^2^).

### 4.9. Measurement of Adenosine Triphosphate (Atp) Content

Cardiac ATP content was measured using an enhanced ATP assay kit (Cat. No. S0027, Beyotime Biotechnology, Shanghai, China) according to the manufacturer’s instructions. Briefly, cardiac tissues were homogenized on ice, boiled, and centrifuged, and the supernatants were collected for ATP detection. ATP working solution was added to the assay wells, followed by the prepared samples, and relative light units were measured using a luminometer to determine ATP levels [68].

### 4.10. RT-qPCR Measurement

Total RNA was extracted from mouse heart tissues using TRIzol reagent (Cat. No. 15596026, Thermo Fisher Scientific, Waltham, MA, USA) according to the manufacturer’s instructions [69]. RNA concentration and purity were assessed using a NanoDrop 2000 spectrophotometer, Thermo Fisher Scientific. Complementary DNA was synthesized by reverse transcription, and quantitative real-time PCR was performed using TaqPro Universal SYBR qPCR Master Mix (Cat. No. Q711-02, Vazyme Biotech Co., Ltd., Nanjing, China) on a real-time PCR system. Relative gene expression was calculated using the 2^^−ΔΔCt^ method with Gapdh as the internal reference. All reactions were performed in triplicate, and amplification specificity was confirmed by melt curve analysis. To further characterize mitochondrial molecular responses, the expression of mitochondrially encoded respiratory-chain gene mitochondrial cytochrome c oxidase subunit I (*mt-Cox1*), mitochondrial cytochrome b (*mt-Cytb*), mitochondrial NADH dehydrogenase subunit 1 (*mt-Nd1*), and SURF1 cytochrome c oxidase assembly factor (*Surf1*) was additionally quantified. The primer pairs used for amplification of the target genes are listed in Supplementary Table S2.

### 4.11. Cell Culture and Drug Treatment

H9c2 rat cardiomyoblasts were obtained from the Cell Resource Center of the Chinese Academy of Medical Sciences and Peking Union Medical College (Beijing, China). Cells were maintained in high-glucose DMEM (Gibco, Thermo Fisher Scientific, Waltham, MA, USA) supplemented with 10% fetal bovine serum (Gibco, Thermo Fisher Scientific, Waltham, MA, USA) and 1% penicillin (Gibco, Thermo Fisher Scientific, Waltham, MA, USA)–streptomycin at 37 °C in a humidified atmosphere containing 5% CO_2_. Cel was dissolved in DMSO to prepare a 1 mM stock solution, whereas DOX was dissolved in normal saline to prepare a 1 mM stock solution. Cells at 70–80% confluence were divided into three groups: Ctrl, DOX, and DOX + Cel. Cells in the Ctrl group received the corresponding vehicle treatment containing equivalent amounts of DMSO and/or normal saline. Cells in the DOX group were treated with 1 μM DOX for 24 h. Cells in the DOX + Cel group were pretreated with 1 μM Cel for 2 h, followed by treatment with 1 μM DOX for 24 h [25].

Mitochondrial reactive oxygen species (mtROS) were assessed using a MitoSOX Red fluorescent probe kit (Cat. No. S0061, Beyotime Biotechnology, China). After treatment, cells were washed with pre-cooled PBS and incubated with 5 μM MitoSOX Red working solution for 10–20 min at 37 °C in the dark. After incubation, cells were washed three times with serum-free medium, and fluorescence signals were detected using a BD FACSCalibur flow cytometer (BD Biosciences, San Jose, CA, USA). Flow-cytometry data were quantitatively analyzed using FlowJo™ software (version 10.8.1; BD Biosciences, Ashland, OR, USA).

### 4.12. Gut Microbiota Analysis

Total genomic DNA was extracted from mouse fecal samples using the FastPure Stool DNA Isolation Kit (MJYH, Shanghai, China) according to the manufacturer’s instructions. The V3–V4 regions of the bacterial 16S rRNA gene were amplified, purified after 2% agarose gel electrophoresis, and quantified using a Qubit 4.0 fluorometer (Thermo Fisher Scientific, Waltham, MA, USA). Sequencing libraries were prepared using the NEXTFLEX Rapid DNA-Seq Kit and sequenced on the Illumina NextSeq 2000 platform (Illumina, Inc., San Diego, CA, USA) by Majorbio Bio-Pharm Technology Co., Ltd. (Shanghai, China).

Amplicon sequence variants were taxonomically assigned against the SILVA 16S rRNA database (v138) using the Naive Bayes classifier implemented in QIIME2 (version 2020.2). Alpha diversity indices, including Chao1 and Shannon, were calculated using mothur (v1.30.1) and compared using the Kruskal–Wallis test followed by Dunn’s multiple-comparisons test [70]. Differentially abundant taxa were identified using LEfSe with thresholds of LDA score > 2 and *p* < 0.05 [71]. Taxa with Spearman’s correlation coefficient > 0.6 and *p* < 0.05 were included in correlation network analysis [72].

### 4.13. Statistical Analysis

Prior to statistical comparison, the normality of data distribution was evaluated using the Shapiro–Wilk test, and homogeneity of variance was assessed by Levene’s test. Student’s *t*-test (or Welch’s *t*-test for unequal variance) was applied for two-group comparisons. One-way ANOVA followed by Tukey’s post hoc test was used for multiple-group pairwise comparisons. For datasets violating parametric assumptions, non-parametric tests were adopted accordingly. Statistical analyses were performed using GraphPad Prism 10 software (GraphPad Software, San Diego, CA, USA). Data are presented as the mean ± standard error of the mean (SEM). A two-tailed *p* < 0.05 was considered statistically significant.

## 5. Conclusions

**Figure 8:**
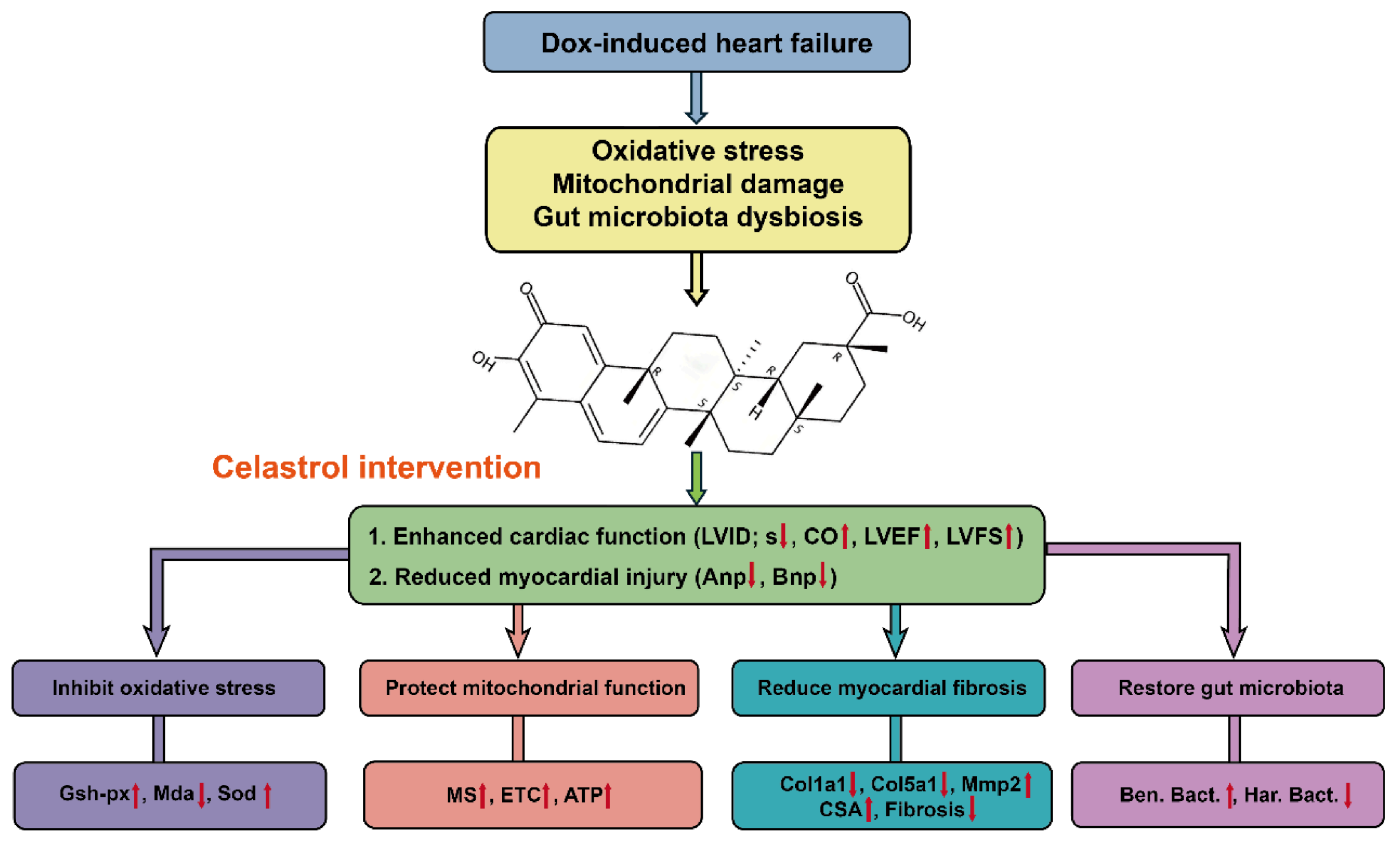
Schematic illustration of the protective effects of Cel against DOX-induced heart failure. DOX induces heart failure characterized by mitochondrial structural damage, enhanced oxidative stress, and gut microbiota dysbiosis. Cel intervention suppresses oxidative stress and enhances antioxidant enzyme activity, preserves mitochondrial integrity, supports respiratory chain-related genes expression, sustains ATP production, and attenuates myocardial fibrosis. Concurrently, Cel restores intestinal microbial homeostasis by promoting the enrichment of beneficial taxa and inhibiting the expansion of inflammation-associated bacteria, thereby collectively mitigating DOX-related cardiotoxicity and preventing the progression of heart failure. Abbreviations: LVIDs, left ventricular internal diameter at systole; CO, cardiac output; LVEF, left ventricular ejection fraction; LVFS, left ventricular fractional shortening; ANP, atrial natriuretic peptide; BNP, B-type natriuretic peptide; Gsh-px, glutathione peroxidase; Mda, malondialdehyde; Sod, superoxide dismutase; MS, mitochondrial structure; ETC, electron transport chain; ATP, adenosine triphosphate; Col1a1, collagen type I alpha 1 chain; Col5a1, collagen type V alpha 1 chain; Mmp2, matrix metallopeptidase 2; CSA, cross-sectional area; Ben. Bact., beneficial bacteria; Har. Bact., harmful bacteria. Red upward arrows indicate increased or improved parameters, whereas red downward arrows indicate decreased parameters.

This study demonstrates that Cel attenuates DOX-induced myocardial injury, preserves cardiac function, and was associated with partial changes in gut microbial diversity and composition. Mechanistically, Cel enhanced antioxidant defense by increasing SOD and GSH-Px activities and reducing MDA accumulation, thereby alleviating DOX-induced oxidative stress. Cel also preserved mitochondrial ultrastructure, supported mitochondrial respiratory chain-related gene expression, and maintained ATP production, suggesting improved mitochondrial bioenergetic homeostasis. In addition, Cel reduced myocardial fibrosis by decreasing collagen deposition and downregulating fibrosis-related genes, including *Col1a1* and *Col5a1*, thereby limiting pathological cardiac remodeling (Figure 8). Furthermore, Cel increased gut microbial diversity, as reflected by elevated Shannon and Chao indices, and enriched beneficial commensal taxa, including *Akkermansia*, *Muribaculaceae*, and *Lachnospiraceae*, suggesting that the amelioration of DOX-induced gut dysbiosis occurred.

Collectively, these findings indicate that Cel attenuates DOX-induced cardiotoxicity through improvements in redox homeostasis, mitochondrial integrity and bioenergetics, and pathological cardiac remodeling. These cardioprotective effects were accompanied by alterations in gut microbial diversity and composition, although the causal contribution of the gut microbiota remains to be established. This study provides experimental evidence supporting the role of Cel as a potential adjuvant candidate for mitigating DOX-associated cardiac injury and gut dysbiosis, and offers a pharmacological basis for further exploring the bioactive constituents of *Tripterygium wilfordii* Hook. f. in modern cardiovascular research.

## Data Availability

The original contributions presented in this study are included in the article/Supplementary Material. Further inquiries can be directed to the corresponding authors.

## Supplementary Materials

The following supporting information can be downloaded at https://www.mdpi.com/article/10.3390/ph19091494/s1, Table S1: Summary of previously reported in vivo celastrol dosing regimens, pharmacological efficacy, and safety/tolerability observations; Table S2: Primers used for the PCR assays in this study.

## Abbreviations

The following abbreviations are used in this manuscript:

ATP	adenosine triphosphate
*Anp*	atrial natriuretic peptide
*Bnp*	brain natriuretic peptide
BW	body weight
Cel	*Celastrol*
CK	creatine kinase
CSA	cross-sectional area
CO	cardiac output
*Col1a1*	type I collagen α1 chain
*Col5a1*	type V collagen α1 chain
DOX	Doxorubicin
ETC	electron transport chain
FTIR	Fourier transform infrared spectroscopy
GSH-Px	glutathione peroxidase
HW	heart weight
HPLC	high-performance liquid chromatography
HE	hematoxylin–eosin
LDH	lactic dehydrogenase
LVEF	left ventricular ejection fraction
LVFS	left ventricular fraction shortening
LVIDs	left ventricular internal dimension at systole
MDA	malondialdehyde
OXPHOS	oxidative phosphorylation
ROS	reactive oxygen species
Sod	superoxide dismutase
TEM	transmission electron microscopy
TL	tibia length
WGA	wheat germ agglutinin
