# Supplementary material for: Celastrol Attenuates Doxorubicin-Induced Heart Failure by Preserving Mitochondrial Integrity and Suppressing Oxidative Stress and Is Accompanied by Changes in Gut Microbiota
Source: Pharmaceuticals (Basel). 2026 Sep 20;19(9):1494. doi: 10.3390/ph19091494 (PMC13610199; doi:10.3390/ph19091494)
Supplement: Supplementary file 1 [file pharmaceuticals-19-01494-s001.zip › Supplementary Table S1.pdf]

Supplementary Table S1. Summary of previously reported in vivo celastrol dosing regimens, pharmacological efficacy, and safety/tolerability observations

Reference	Animal Species	Strain	Sex	Model	Model Induction	Celastrol Dose	Dosing Frequency	Duration	Affected Outcomes	Unaffected Outcomes	Protective/Toxic Effect
Int J Ophthalmol. 2022;15(8):1221–1230	Mouse	C57BL/6J	NR	Laser-induced choroidal neovascularization (CNV)	Four laser photocoagulation spots per eye to induce CNV	0.1, 0.5 mg/kg/day	Once daily	14 days	0.1 mg/kg: CNV area ↓ ~49.15%; 0.5 mg/kg: CNV area ↓ ~80.26%, markedly stronger than 0.1 mg/kg; fluorescence leakage ↓; VEGF-related angiogenesis ↓	No normal mouse + celastrol-only group	Protective/anti-angiogenic effect.
PLoS One. 2012;7(4):e35733	Mouse	BALB/c	NR	Normal-animal hematopoietic/immunotoxicity model	No disease model; normal mice treated directly	0.01, 0.1, 1, 5 mg/kg/day	Once daily	4 days	5 mg/kg/day: clinical toxicity including hunched posture and ruffled fur; impaired B-cell and erythroid development; bone-marrow CLP, CMP, and MEP ↓; abnormalities in splenic and bone-marrow hematopoiesis	At 0.01, 0.1, and 1 mg/kg, routine peripheral RBC/HGB/HCT did not show abnormalities comparable to those at 5 mg/kg	Toxic effect; 5 mg/kg caused clear immune/hematopoietic toxicity; ≤1 mg/kg provides only short-term indirect tolerability evidence.
Cancer Res. 2019;79(3):685	Mouse	BALB/cA nude	Male	PC-3 human prostate cancer xenograft	PC-3 cells injected subcutaneously; treatment started when tumors reached ~130 mm^3	0.5, 2 mg/kg/day	Once daily	16 days	0.5 mg/kg/day: only a modest effect on tumor growth; 2 mg/kg/day: tumor volume, tumor weight, and angiogenesis significantly ↓	No significant change in body weight at 2 mg/kg	Antitumor/anti-angiogenic effect; 0.5 mg/kg showed weaker efficacy.
Diabetol Metab Syndr. 2026;18(1):55	Mouse	db/db; normal control db/m	Male	Diabetic kidney disease (DKD)	Genetic dub/db diabetes model	0.25, 0.5, 1 mg/kg/day	Once daily	8 weeks	Celastrol improved Scr, 24-h proteinuria, blood lipids, and renal histopathology; GSH and SOD ↑; MDA and iron accumulation ↓; 1 mg/kg produced the strongest overall effect and was used as a key dose in subsequent experiments; 0.5 mg/kg was also therapeutic	No normal db/m + 0.5 mg/kg celastrol-only group	Renoprotective effect; 0.5 mg/kg was an effective dose.
Pediatr Res. 2024;96(7):1681–1692	Rat	Sprague-Dawley	Male	Neonatal hypoxic-ischemic brain injury	Common carotid artery procedure + 8% O_2 hypoxia for 2.5 h	0.5, 1, 2 mg/kg	Post-hypoxia dosing, approximately once every 24 h	Dose screening and subsequent observation	0.5 mg/kg showed some protection; 1 mg/kg produced the greatest reduction in cerebral infarction and was therefore used in subsequent experiments; at 1 mg/kg, infarction, cerebral edema, microglial activation, inflammation, and oxidative stress ↓	0.5 mg/kg was less effective than 1 mg/kg; no normal P7 rat + 0.5 mg/kg systemic toxicology group	Neuroprotective effect; 1 mg/kg showed the best efficacy.
Acta Biochim Biophys Sin (Shanghai). 2022;54(8):1180–1192	Mouse	C57BL/6J	Male	Direct intervention in normal animals/cardiotoxicity	Normal mice treated directly	1 mg/kg/day; 2 mg/kg/day; 4 mg/kg every other day	1 and 2 mg/kg daily; 4 mg/kg every other day	2 weeks	2 mg/kg/day and 4 mg/kg every other day: heart weight ↓, HW/TL ↓, EF ↓, FS ↓, LVIDs ↑; myocardial hypertrophy/fibrosis ↑; in NRVMs, viability ↓, apoptosis ↑, mitochondrial membrane potential ↓, Bcl-2 ↓, Bax/Caspase-3/12 ↑, and ER stress/CHOP ↑	1 mg/kg/day for 2 weeks: no significant changes in heart weight, major cardiac structure, or systolic function	Toxic effect; no obvious short-term cardiotoxicity at 1 mg/kg.
Int J Pharmacol. 2023;19(1):89–99	Rat	Wistar	Male	Isoproterenol (ISO)-induced myocardial injury/heart failure	ISO 85 mg/kg i.p. on days 6 and 7	5 mg/kg/day	Once daily	7 days	5 mg/kg: heart weight/body weight ratio ↓, infarct size ↓; hemodynamics improved; LDH, CK-MB, and TnT ↓; antioxidant enzymes ↑ and MDA ↓; TNF-α, IL-6, and NF-κB ↓; Bax/Caspase-3 ↓ and Bcl-2 ↑	In the celastrol-only 5 mg/kg group, most parameters were comparable to normal controls, with no obvious cardiac injury	Protective effect.
Iran J War Public Health. 2023;15(4):361–367	Mouse	Swiss albino	NR	Sepsis-induced myocardial injury	Cecal ligation and puncture (CLP)	2 mg/kg	Single dose	1 h before CLP	IL-6, TNF-α, TLR4, F2-isoprostane, cTn, and CK-MB ↓; IL-10 improved; myocardial histopathological injury attenuated	Organ toxicity in normal mice at 2 mg/kg was not systematically reported	Protective effect.
Cell Stress Chaperones. 2015;20(1):185–201	Mouse	C57BL/6J	Male	Doxorubicin (DOX)-induced cardiomyopathy	DOX 20 mg/kg, single i.p. injection	4 mg/kg	Approximately once daily	~7 days; first dose 12 h before DOX	LV function improved; remodeling, apoptosis, and inflammation ↓; HSP70 ↑	Celastrol-only mice showed no overt clinical toxicity and no macroscopic/microscopic injury in the major organs examined.	Protective effect.
Cell Stress Chaperones. 2015;20(1):185–201	Mouse	C57BL/6J	NR	Acute myocardial infarction	Coronary artery ligation	4 mg/kg	Approximately once daily	28 days	Left ventricular function improved; infarct size, apoptosis, and inflammation ↓	No overt toxicity was reported for celastrol treatment	Protective effect.
Acta Pharm Sin B. 2022;12(5):2300–2314	Mouse	C57BL/6	Male	CCl4-induced hepatic fibrosis	CCl4 0.5 mL/kg i.p. twice weekly for 6 weeks	0.25, 0.5, 1 mg/kg/day	Once daily	Last 3 weeks	Dose-dependent improvement at 0.25–1 mg/kg; ALT, AST, ALP, collagen, hydroxyproline, α-SMA, VIM, and COL1A1 ↓; effects were more pronounced at 0.5–1 mg/kg	No normal celastrol-only group; organ toxicity cannot be excluded at these doses	Protective/antifibrotic effect.
Aging (Albany NY). 2017;9(10):2069–2082	Mouse	C57BL/6N	Male	Diet-induced obesity (DIO)	60 kcal% high-fat diet for 12 weeks	5, 7.5 mg/kg/day	Once daily	21 days	5 mg/kg: body weight and adiposity ↓; glucose tolerance and insulin sensitivity improved; IL-6, IL-1β, and M1 markers ↓; overall effects were stronger at 7.5 mg/kg	No clear signal for plasma TNF-α; no normal celastrol-only long-term toxicity group	Protective effect.
Diabetol Metab Syndr. 2023;15(1):186	Mouse	C57BL/6	Male	Diabetic cardiomyopathy	STZ 50 mg/kg i.p. for 5 days	1 mg/kg every 2 days	Every 2 days	16 weeks	CK-MB and LDH ↓; LVEDD/LVESD improved; EF/FS ↑; fibrosis, inflammation, ROS, and apoptosis ↓	No normal mouse long-term celastrol-only group	Protective effect.
BioMed Res Int. 2023;2023:1076522	Mouse	C57BL/6 background	Male	Acute ischemic stroke	Permanent middle cerebral artery occlusion (MCAO)	1 mg/kg	Immediately after surgery and once on day 1	Two doses; evaluated on day 3	Infarct volume, apoptosis, TNF-α, IL-1β, IL-18, NLRP3, and Caspase-1 ↓; Nrf2/HO-1 ↑	No normal long-term celastrol-only group	Protective effect.
Oncotarget. 2017;8(40):67300–67314	Mouse	C57BL/6 background	NR	LPS-induced systemic inflammation/septic shock	LPS i.p.	1 mg/kg	Single dose 1 h before LPS	Short-term	IL-1β, Caspase-1, and NLRP3 ↓; splenic inflammation improved; survival ↑	In some cell experiments, TNF-α and IL-6 showed no significant changes; no systematic toxicity evaluation	Protective effect.
	Mouse	C57BL/6 background	Male	DSS-induced colitis	3% or 5% DSS in drinking water for 7 days	1 mg/kg	Once daily	Started 2 days before DSS; ~10–12 days	DAI, hematochezia, and histopathology improved; cleaved Caspase-1, NLRP3, ASC, and IL-1β ↓	No systematic organ toxicity evaluation	Protective effect.
Cell Biochem Biophys. 2022;80(1):191–202	Rat	Swiss albino rat	Male	Renal ischemia-reperfusion injury (IRI)	Left nephrectomy + right renal artery clamping for 30 min, followed by 24 h reperfusion	2 mg/kg/day	Once daily	7 days before IRI	Scr, BUN, MDA, KIM-1, IL-6, TNF-α, NF-κB, p-ERK, and Caspase-3 ↓; GSH, Nrf2/HO-1, and PI3K/AKT ↑	In the Sham + celastrol 2 mg/kg group, Scr, MDA, GSH, and related indices did not differ significantly from Sham; cautiously suggests no obvious renal toxicity after oral 2 mg/kg for 7 days	Protective effect; short-term renal safety information.

Reference	Animal Species	Strain	Sex	Model	Model Induction	Celastrol Dose	Dosing Frequency	Duration	Affected Outcomes	Unaffected Outcomes	Protective/Toxic Effect
J Cell Mol Med. 2020;24(1):941–953	Rat	Sprague-Dawley	Male	CCl4-induced hepatic fibrosis	CCl4 and olive oil (1:1) every other day for 8 weeks	0.25, 0.5, 1 mg/kg/day	Once daily	Weeks 5–8; 4 weeks total	0.25 mg/kg produced partial improvement; effects increased at 0.5 mg/kg and were strongest at 1 mg/kg: liver enzymes, collagen, fibrosis, and inflammatory markers ↓; p-AMPK/SIRT3 ↑	No normal celastrol-only group	Protective effect.
Pharmaceutics. 2026;18(6):695	Mouse	DBA/1JGpt	Male	Collagen-induced arthritis (CIA)	Type II collagen + CFA, with booster on day 21	2 mg/kg	Once daily	30 days	Free celastrol reduced paw swelling by ~22.79%; Cel-SMEDDS reduced it by ~37.42%; arthritis score, TNF-α, and cartilage/bone damage ↓	After Cel-SMEDDS treatment, arthritis scores did not differ significantly from normal mice; this was not a systemic toxicology assessment	Protective effect.
	Mouse	KM	Female	Normal-animal safety study	Normal mice treated directly	16 mg/kg/day	Once daily	27 days	Slightly slower body-weight gain	16 mg/kg/day for 27 days: no obvious clinical toxicity; hematology and liver/kidney function were generally not significantly altered; no obvious histological damage in major organs	Preliminary safety evidence.
Chin Med. 2026;21(1):149	Mouse	ApoE-/-; C57BL/6 background	Male	Atherosclerosis	ApoE-/- + high-fat diet	0.25, 0.5, 1 mg/kg every 2 days	Every 2 days	12 weeks	All doses (0.25–1 mg/kg) showed anti-atherosclerotic effects: plaque and lipid burden ↓; collagen ↑; TG and LDL-C ↓; TLR4, p-STAT3, NLRP3, Caspase-1, N-GSDMD, and IL-1β ↓	HDL-C showed no significant change; no normal celastrol-only group	Protective effect.
Mol Med. 2025;31(1):233	Mouse	C57BL/6J	Male	DIO-associated endothelial dysfunction	45 kcal% high-fat diet for 8 weeks	0.1 mg/kg/day	Once daily	8 weeks	Body weight, TC, TG, blood pressure, and blood glucose improved; hepatic lipid accumulation ↓; ACh-EDR, NO, and eNOS ↑; ROS and ER stress ↓; AMPK ↑	PE-induced contraction showed no significant difference; SNP-mediated endothelium-independent relaxation showed no significant difference; findings cannot be extrapolated to indicate absence of systemic toxicity	Protective effect.
PLoS One. 2013;8(3):e58391	Mouse	Nude mouse	NR	VCaP prostate cancer xenograft	4 × 10^6 VCaP cells injected subcutaneously	0.5 mg/kg	4 times per week	3 weeks	Tumor growth ↓; AR, ERG, and p-NF-κB p65 ↓	No significant body-weight loss; complete necropsy showed no significant toxicity in lung, kidney, liver, spleen, or heart	Antitumor effect; no obvious short-term toxicity.
PLoS One. 2010;5(12):e14153	Mouse	Nude mouse	Female	PC-3 prostate cancer xenograft	Subcutaneous PC-3 tumor implantation	1 mg/kg	Continuous	3 weeks	Tumor growth, Ki67, and CD31 ↓; TUNEL and IκBα ↑; NF-κB target genes ↓	Authors described minimal systemic toxicity; no systematic organ toxicology assessment	Antitumor effect; limited systemic toxicity.
Brain Res. 2015;1609:21–30	Rat	NR	NR	Optic nerve crush injury	Optic nerve clamped for 5 s	Systemic 1 mg/kg/day; intravitreal 0.2, 1, 5 mg/kg	i.p. daily; intravitreal single dose	2 weeks	Systemic 1 mg/kg/day i.p. significantly increased RGC survival; intravitreal 1 and 5 mg/kg were effective; TNF-α ↓	Intravitreal 0.2 mg/kg showed no statistically significant protection; HO-1, Hsf1, and Hsp70 showed no significant changes	Neuroprotective effect; evidence of a dose threshold.
Cell Death Dis. 2018;9(6):601	Mouse	BALB/c	Male	UUO-induced renal fibrosis	Left ureteral ligation	1 mg/kg/day	Once daily	7 days	Tubular injury, collagen, fibronectin, collagen I, α-SMA, Vim, Col1a1, and TGF-β1 ↓; CB2R ↑	No normal celastrol-only group	Protective effect.
	Mouse	BALB/c	Male	Folic acid-induced renal fibrosis	FA 250 mg/kg i.p., single dose	1 mg/kg/day	Once daily	21 days	Renal fibrosis, ECM deposition, and fibrosis markers ↓; CB2R ↑	No normal long-term celastrol-only group	Protective effect.
Acta Pharmacol Sin. 2021;42(9):1472–1485	Mouse	Nude mouse	Male	ccRCC xenograft + high-fat diet	786-O xenograft	0.25, 0.5, 1 mg/kg/day	Once daily	4 weeks	0.25, 0.5, and 1 mg/kg dose-dependently inhibited tumor growth; LXRα/ABCA1 ↑; lipid accumulation ↓	Insufficient multi-organ toxicology data in normal mice	Antitumor/protective effect.
Acta Pharmacol Sin. 2023;44(8):1637–1648	Mouse	C57BL/6J	Male	HCV NS5B-induced hepatic inflammation	pAde-NS5B via tail vein	0.2, 0.5 mg/kg	Twice daily	5 days	Both 0.2 and 0.5 mg/kg attenuated inflammation, with stronger effects at 0.5 mg/kg; CD68, Nlrp3, AST, ALT, IFN-γ, MCP-1, and IL-1β ↓	Masson staining showed no scarring; no normal celastrol-only systemic toxicology group	Protective effect.
Int J Mol Sci. 2026;27(9):3849	Rat	SHR; control WKY	Male	Spontaneous hypertension-associated renal injury	Genetic SHR model	0.5, 1 mg/kg/day	Once daily	6 weeks	0.5 mg/kg produced improvement; 1 mg/kg was more effective: RAAS, inflammation, and MDA ↓; SOD, GSH-Px, CAT, Nrf2/NQO1/HO-1 ↑	No normal WKY + celastrol group	Protective effect.
Br J Pharmacol. 2014;171(23):5265–5279	Rat	Sprague-Dawley CD CRL	Female	Myocardial infarction	Permanent LAD ligation	1 mg/kg/day	Once daily	14 days	Cardiac function ↑; LV remodeling, infarction, fibrosis, macrophage infiltration, TGF-β, and collagen ↓; HO-1 ↑	Sham + celastrol and a separate normal-rat pilot were included. At 1 mg/kg/day i.p. for 14 days, all animals survived; general serum biochemistry markers of liver, kidney and heart health showed no significant differences in the pilot	Cardioprotective effect; preliminary safety evidence.

Table note. ↑, increased; ↓, decreased. NR is used only when the information could not be confirmed in the original article.

Abbreviations: NR, not reported; i.p., intraperitoneal; s.c., subcutaneous; p.o., oral administration; CNV, choroidal neovascularization; VEGF, vascular endothelial growth factor; RBC, red blood cell; HGB, hemoglobin; HCT, hematocrit; CLP, common lymphoid progenitor; CMP, common myeloid progenitor; MEP, megakaryocyte–erythroid progenitor; SHR, spontaneously hypertensive rat; WKY, Wistar–Kyoto rat; Ang II, angiotensin II; ACE, angiotensin-converting enzyme; Scr, serum creatinine; TNF-α, tumor necrosis factor-α; IL, interleukin; MDA, malondialdehyde; SOD, superoxide dismutase; GSH-Px, glutathione peroxidase; CAT, catalase; Nrf2, nuclear factor erythroid 2-related factor 2; NQO1, NAD(P)H quinone dehydrogenase 1; HO-1, heme oxygenase-1; DKD, diabetic kidney disease; GSH, glutathione; HW/TL, heart weight/tibial length ratio; EF, ejection fraction; FS, fractional shortening; LVIDs, left ventricular internal diameter at end-systole; NRVMs, neonatal rat ventricular myocytes; ER, endoplasmic reticulum; CHOP, C/EBP homologous protein; ISO, isoproterenol; LDH, lactate dehydrogenase; CK-MB, creatine kinase-MB; TnT, troponin T; NF-κB, nuclear factor kappa B; TLR4, Toll-like receptor 4; cTn, cardiac troponin; DOX, doxorubicin; LV, left ventricular; HSP70, heat shock protein 70; CCl4, carbon tetrachloride; ALT, alanine aminotransferase; AST, aspartate aminotransferase; ALP, alkaline phosphatase; α-SMA, alpha-smooth muscle actin; VIM, vimentin; COL1A1, collagen type I alpha 1 chain; DIO, diet-induced obesity; STZ, streptozotocin; LVEDD, left ventricular end-diastolic diameter; LVESD, left ventricular end-systolic diameter; ROS, reactive oxygen species; MCAO, middle cerebral artery occlusion; NLRP3, NOD-like receptor family pyrin domain-containing 3; IRI, ischemia–reperfusion injury; BUN, blood urea nitrogen; KIM-1, kidney injury molecule-1; PI3K, phosphoinositide 3-kinase; AKT, protein kinase B; ERK, extracellular signal-regulated kinase; CIA, collagen-induced arthritis; CFA, complete Freund's adjuvant; Cel-SMEDDS, celastrol self-microemulsifying drug delivery system; ApoE−/−, apolipoprotein E knockout; TG, triglyceride; LDL-C, low-density lipoprotein cholesterol; STAT3, signal transducer and activator of transcription 3; GSDMD, gasdermin D; HDL-C, high-density lipoprotein cholesterol; AMPK, AMP-activated protein kinase; SIRT3, sirtuin 3; TC, total cholesterol; ACh-EDR, acetylcholine-induced endothelium-dependent relaxation; NO, nitric oxide; eNOS, endothelial nitric oxide synthase; PE, phenylephrine; SNP, sodium nitroprusside; AR, androgen receptor; ERG, ETS-related gene; RGC, retinal ganglion cell; HSF1, heat shock factor 1; UUO, unilateral ureteral obstruction; TGF-β1, transforming growth factor-β1; CB2R, cannabinoid receptor type 2; FA, folic acid; ECM, extracellular matrix; ccRCC, clear cell renal cell carcinoma; LXRα, liver X receptor alpha; ABCA1, ATP-binding cassette transporter A1; HCV, hepatitis C virus; IFN-γ, interferon-γ; MCP-1, monocyte chemoattractant protein-1; RAAS, renin–angiotensin–aldosterone system; LAD, left anterior descending coronary artery.

References for Supplementary Table S1

[16] Li, Z., Zhou, K. W., Chen, F., Shang, F., & Wu, M. X. (2022). Celastrol inhibits laser-induced choroidal neovascularization by decreasing VEGF induced proliferation and migration. *International journal of ophthalmology*, *15*(8), 1221–1230. https://doi.org/10.18240/ijo.2022.08.01

[17] Kusy, S., Ghosn, E. E., Herzenberg, L. A., & Contag, C. H. (2012). Development of B cells and erythrocytes is specifically impaired by the drug celastrol in mice. *PloS one*, *7*(4), e35733. https://doi.org/10.1371/journal.pone.0035733

[18] Deng, Y., Wang, J., Liu, X., Wang, X., Li, H., Gu, B., Zhang, M., Wang, R., & Yang, Y. (2026). Celastrol Ameliorates Renal Injury in Spontaneously Hypertensive Rats by Activating the Nrf2/Ho-1 Signaling Pathway to Alleviate Oxidative Stress. *International journal of molecular sciences*, *27*(9), 3849. https://doi.org/10.3390/ijms27093849

[19] Pang, X.; Yi, Z.; Zhang, J.; Lu, B.; Sung, B.; Qu, W.; Aggarwal, B.B.; Liu, M. Correction: Celastrol Suppresses Angiogenesis-Mediated Tumor Growth through Inhibition of AKT/Mammalian Target of Rapamycin Pathway. *Cancer Res.* **2019**, *79*, 685. https://doi.org/10.1158/0008-5472.Can-18-3859

[20] Tang, Y. W., Xu, T. J., Cao, J. W., Jiang, M. Y., Yang, R. C., & Wan, F. (2026). Celastrol mitigates diabetic kidney disease by inhibiting ferroptosis via the SIRT1/NRF2/GPX4 pathway. *Diabetology & metabolic syndrome*, *18*(1), 55. https://doi.org/10.1186/s13098-026-02085-4

[21] Hu, Y., Nan, Y., Lin, H., Zhao, Q., Chen, T., Tao, X., Ding, B., Lu, L., Chen, S., Zhu, J., Guo, X., & Lin, Z. (2024). Celastrol ameliorates hypoxic-ischemic brain injury in neonatal rats by reducing oxidative stress and inflammation. *Pediatric research*, *96*(7), 1681–1692. https://doi.org/10.1038/s41390-024-03246-9

[22] Chen, Z., Zhuang, Z., Meng, C., Zhu, Z., Zhang, Y., & Zhang, Z. (2022). Induction of the ER stress response in NRVMs is linked to cardiotoxicity caused by celastrol. *Acta biochimica et biophysica Sinica*, *54*(8), 1180–1192. https://doi.org/10.3724/abbs.2022104

[23] Lian, W., Liu, S., Li, Y., Wang, L., & Gong, J. (2023). Celastrol improves isoproterenol-induced heart failure by reducing inflammation, apoptosis and oxidative stress. *International Journal of Pharmacology*, *19*(1), 89-99.

[24] Ghafil, F. A., Hassan, E. S., Aziz, N. D., Salim, M. M., Majeed, S. A., Rasheed, S. M. H., & Mardan, H. W. (2023). Cardioprotective potential of celastrol in sepsis-induced cardiotoxicity; Mouse model of endotoxemia. *Iranian Journal of War and Public Health*, *15*(4), 361-367.

[25] Sharma, S., Mishra, R., Walker, B. L., Deshmukh, S., Zampino, M., Patel, J., Anamalai, M., Simpson, D., Singh, I. S., Kaushal, S., & Kaushal, S. (2015). Celastrol, an oral heat shock activator, ameliorates multiple animal disease models of cell death. *Cell stress & chaperones*, *20*(1), 185–201. https://doi.org/10.1007/s12192-014-0536-1

[26] Luo, P., Liu, D., Zhang, Q., Yang, F., Wong, Y. K., Xia, F., Zhang, J., Chen, J., Tian, Y., Yang, C., Dai, L., Shen, H. M., & Wang, J. (2022). Celastrol induces ferroptosis in activated HSCs to ameliorate hepatic fibrosis *via* targeting peroxiredoxins and HO-1. *Acta pharmaceutica Sinica. B*, *12*(5), 2300–2314. https://doi.org/10.1016/j.apsb.2021.12.007

[27] Luo, D., Guo, Y., Cheng, Y., Zhao, J., Wang, Y., & Rong, J. (2017). Natural product celastrol suppressed macrophage M1 polarization against inflammation in diet-induced obese mice via regulating Nrf2/HO-1, MAP kinase and NF-κB pathways. *Aging*, *9*(10), 2069–2082. https://doi.org/10.18632/aging.101302

[28] Zhao, X., Huang, B., Zhang, J., Xiang, W., & Zhu, N. (2023). Celastrol attenuates streptozotocin-induced diabetic cardiomyopathy in mice by inhibiting the ACE / Ang II / AGTR1 signaling pathway. *Diabetology & metabolic syndrome*, *15*(1), 186. https://doi.org/10.1186/s13098-023-01159-x

[29] Cao, F., Wang, Y., Song, Y., Xu, F., Xie, Q., Jiang, M., Liu, X., Zhang, D., & Xu, L. (2023). Celastrol Treatment Ameliorated Acute Ischemic Stroke-Induced Brain Injury by Microglial Injury Inhibition and Nrf2/HO-1 Pathway Activations. *BioMed research international*, *2023*, 1076522. https://doi.org/10.1155/2023/1076522

[30] Yu, X., Zhao, Q., Zhang, X., Zhang, H., Liu, Y., Wu, X., Li, M., Li, X., Zhang, J., Ruan, X., & Zhang, H. (2017). Celastrol ameliorates inflammation through inhibition of NLRP3 inflammasome activation. *Oncotarget*, *8*(40), 67300–67314. https://doi.org/10.18632/oncotarget.18619

[31] Younis, N. S., & Ghanim, A. M. H. (2022). The Protective Role of Celastrol in Renal Ischemia-Reperfusion Injury by Activating Nrf2/HO-1, PI3K/AKT Signaling Pathways, Modulating NF-κb Signaling Pathways, and Inhibiting ERK Phosphorylation. *Cell biochemistry and biophysics*, *80*(1), 191–202. https://doi.org/10.1007/s12013-022-01064-6

[32] Wang, Y., Li, C., Gu, J., Chen, C., Duanmu, J., Miao, J., Yao, W., Tao, J., Tu, M., Xiong, B., Zhao, L., & Liu, Z. (2020). Celastrol exerts anti-inflammatory effect in liver fibrosis via activation of AMPK-SIRT3 signalling. *Journal of cellular and molecular medicine*, *24*(1), 941–953. https://doi.org/10.1111/jcmm.14805

[33] Ma, B., Li, Y., Zhang, J., Fu, Y., & Cao, H. (2026). Orally Administered Self-Microemulsifying Celastrol Alleviates Rheumatoid Arthritis by Modulating the Expression of TNF-α. *Pharmaceutics*, *18*(6), 695. https://doi.org/10.3390/pharmaceutics18060695

[34] Shi, Y., Li, Z., Liu, Q., Qiu, Y., Long, J., Yang, Q., Zhang, C., Gong, Y., & Qin, L. (2026). Celastrol ameliorates atherosclerosis by inhibiting TLR4/STAT3/NLRP3-mediated macrophage pyroptosis. *Chinese medicine*, *21*(1), 149. https://doi.org/10.1186/s13020-026-01430-z

[35] Yu, C., Lin, W., Yang, J., Jiang, Q., Liu, W., Liu, H., Lin, Y., Wang, L., Chen, L., Huang, Y., & Chen, L. (2025). Celastrol improves endothelial function in diet-induced obesity mice via attenuating endoplasmic reticulum stress through the activation of AMPK pathway. *Molecular medicine (Cambridge, Mass.)*, *31*(1), 233. https://doi.org/10.1186/s10020-025-01259-6

[36] Shao, L., Zhou, Z., Cai, Y., Castro, P., Dakhov, O., Shi, P., Bai, Y., Ji, H., Shen, W., & Wang, J. (2013). Celastrol suppresses tumor cell growth through targeting an AR-ERG-NF-κB pathway in TMPRSS2/ERG fusion gene expressing prostate cancer. *PloS one*, *8*(3), e58391. https://doi.org/10.1371/journal.pone.0058391

[37] Dai, Y., Desano, J., Tang, W., Meng, X., Meng, Y., Burstein, E., Lawrence, T. S., & Xu, L. (2010). Natural proteasome inhibitor celastrol suppresses androgen-independent prostate cancer progression by modulating apoptotic proteins and NF-kappaB. *PloS one*, *5*(12), e14153. https://doi.org/10.1371/journal.pone.0014153

[38] Kyung, H., Kwong, J. M., Bekerman, V., Gu, L., Yadegari, D., Caprioli, J., & Piri, N. (2015). Celastrol supports survival of retinal ganglion cells injured by optic nerve crush. *Brain research*, *1609*, 21–30. https://doi.org/10.1016/j.brainres.2015.03.032

[39] Tang, M., Cao, X., Zhang, K., Li, Y., Zheng, Q. Y., Li, G. Q., He, Q. H., Li, S. J., Xu, G. L., & Zhang, K. Q. (2018). Celastrol alleviates renal fibrosis by upregulating cannabinoid receptor 2 expression. *Cell death & disease*, *9*(6), 601. https://doi.org/10.1038/s41419-018-0666-y

[40] Zhang, C. J., Zhu, N., Long, J., Wu, H. T., Wang, Y. X., Liu, B. Y., Liao, D. F., & Qin, L. (2021). Celastrol induces lipophagy via the LXRα/ABCA1 pathway in clear cell renal cell carcinoma. *Acta pharmacologica Sinica*, *42*(9), 1472–1485. https://doi.org/10.1038/s41401-020-00572-6

[41] Chen, S. R., Li, Z. Q., Xu, J., Ding, M. Y., Shan, Y. M., Cheng, Y. C., Zhang, G. X., Sun, Y. W., Wang, Y. Q., & Wang, Y. (2023). Celastrol attenuates hepatitis C virus translation and inflammatory response in mice by suppressing heat shock protein 90β. *Acta pharmacologica Sinica*, *44*(8), 1637–1648. https://doi.org/10.1038/s41401-023-01067-w

[42] Der Sarkissian, S., Cailhier, J. F., Borie, M., Stevens, L. M., Gaboury, L., Mansour, S., Hamet, P., & Noiseux, N. (2014). Celastrol protects ischaemic myocardium through a heat shock response with up-regulation of haeme oxygenase-1. *British journal of pharmacology*, *171*(23), 5265–5279. https://doi.org/10.1111/bph.12838
