# Supplementary material for: Celastrol Attenuates Doxorubicin-Induced Heart Failure by Preserving Mitochondrial Integrity and Suppressing Oxidative Stress and Is Accompanied by Changes in Gut Microbiota
Source: Pharmaceuticals (Basel). 2026 Sep 20;19(9):1494. doi: 10.3390/ph19091494 (PMC13610199; doi:10.3390/ph19091494)
Supplement: Supplementary file 1 [file pharmaceuticals-19-01494-s001.zip › Supplementary Table S2.pdf]

Supplemental Table S2. Primers used for the PCR assays in this study.

Primer	Sequence (5' to 3')
Mouse-*Anp*-forward	GCTTCGGGGGTAGGATTGAC
Mouse-*Anp*-reverse	GAGGCAAGACCCCACTAGAC
Mouse-*Bnp*-forward	GGGCTGTAACGCACTGAAGTT
Mouse-*Bnp*-reverse	TGTGGCAAGTTTGTGCTCCA
Mouse-*Col1a1*-forward	CCTCAGGGTATTGCTGGACAAC
Mouse-*Col1a1*-reverse	CAGAAGGACCTTGTTTGCCAGG
Mouse-*Col5a1*-forward	CTTCGCCGCTACTCCTGTTC
Mouse-*Col5a1*-reverse	CCCTGAGGGCAAATTGTGAAAA
Mouse-*Ndufb8*-forward	GGAATCGTGTGGACACGTCA
Mouse-*Ndufb8*-reverse	GCACGTAGAGGGAAGGACAA
Mouse-*Sdhb*-forward	CGTTCTCGGCAGAGTCGG
Mouse-*Sdhb*-reverse	TCGTACGTCTGCATTCGAGG
Mouse-*Uqcc1*-forward	TTGCTGGTGCGAGTCCTTAG
Mouse-*Uqcc1*-reverse	GTGTCCGCTCCAACAGTCT
Mouse-*Cox6a1*-forward	TCAACGTGTTCCTCAAGTCGC
Mouse-*Cox6a1*-reverse	AGGGTATGGTTACCGTCTCCC
Mouse-*Atp5f1a*-forward	TCCGAGAAGCTGCAAGGATG
Mouse-*Atp5f1a*-reverse	GACATCTCAGCTGTGCCAGT
Mouse-*Mmp2*-forward	ACCTGAACACTTTCTATGGCTG
Mouse-*Mmp2*-reverse	CTTCCGCATGGTCTCGATG
Mouse-Surf1-Forword	CAGCCTGATGGAGAAGATGG
Mouse -Surf1-Reverse	TGCTTCTTCACGTCCAGGTC
Mouse -mt-Nd1-Forword	CGGCCCATTCGCGTTATTC
Mouse -mt-Nd1-Reverse	GATCGTAACGGAAGCGTGGA
Mouse -mt-Cytb-Forword	CTTCGCTTTCCACTTCATCTTACC
Mouse -mt-Cytb-Reverse	TTGGGTTGTTTGATCCTGTTTCG
Mouse -mt-Cox1-Forword	TGCCCAAAGAATCAGAACAG

Mouse -mt-Cox1-Reverse	CTAGCCGCAGGCATTACTAT
m-TNF-α-Forword	CCCTCACACTCAGATCATCTTCT
m-TNF-α-Reverse	GCTACGACGTGGGCTACAG
m-IL-1β-Forword	GAAATGCCACCTTTTGACAGTG
m-IL-1β-Reverse	TGGATGCTCTCATCAGGACAG
m-IL-6-Forword	TAGTCCTTCCTACCCCAATTTCC
m-IL-6-Reverse	TTGGTCCTTAGCCACTCCTTC
